# Widespread Aquatic Insect Responses to Recent Warming in Swiss Mountain Lakes

**DOI:** 10.1111/gcb.70957

**Published:** 2026-06-08

**Authors:** Maja Damber, Ghéreint Devillet, Pierre Lapellegerie, Sandra O. Camara‐Brugger, Laura Jiménez‐Liébanas, Carmen Docci, Juliane Krenz, Nikolaus J. Kuhn, André F. Lotter, Oliver Heiri

**Affiliations:** ^1^ Department of Environmental Sciences University of Basel Basel Switzerland; ^2^ Institute of Plant Sciences University of Bern Bern Switzerland; ^3^ Oeschger Centre for Climate Change Research University of Bern Bern Switzerland

**Keywords:** Chironomidae, environmental impacts, mountain lakes, palaeolimnology, surface sediment, Switzerland

## Abstract

Mountain lakes are highly sensitive to climatic change, yet the extent to which alpine aquatic communities respond to recent warming remains uncertain. We used a palaeolimnological approach based on analysing the remains of non‐biting midge (chironomid) larvae preserved in surface sediments from 24 Swiss mountain lakes to assess species‐environment relationships of chironomids and compare our results to previous, detailed survey data from similar campaigns in 1993/2002. This allowed us to determine changes in chironomid assemblages relative to lake physicochemistry and increasing temperatures over the past decades. We show that high‐elevation oligo‐ to mesotrophic lakes generally experienced a shift towards warmer chironomid assemblages, expansion of taxa with larger thermal range, and a simultaneous reduction in cold‐stenothermic taxa, consistent with rising air and water temperatures. However, 9 out of 24 lakes, mainly in the lower‐elevational range, exhibited stable assemblages or, in some cases, shifts towards colder chironomid communities. This is likely related to local catchment and lake conditions such as shading, changing human activities such as pasturing or hydrological inputs from snow and ice, indicating highly individual, lake‐specific responses. Environmental parameters accounting for the highest variability in the modern distribution of chironomid assemblages include variables representing lakewater organic matter content and elevation, as well as oxygen, total nitrogen, and total phosphorus concentrations, demonstrating the sensitivity of chironomid assemblages to temperature and associated limnological variables. Overall, our findings highlight that both large‐scale climatic drivers and local environmental heterogeneity shape chironomid assemblages in alpine environments, and that the majority of Swiss mountain lakes are showing responses in aquatic insect communities due to increasing temperatures. We conclude that shifts in chironomid populations are expected to further increase in amplitude under continued warming in the Alps, as rising temperatures increasingly affect alpine ecosystems and progressively cross critical thermal thresholds and tipping points.

## Graphical abstract

Chironomids in surface sediment from 24 Swiss mountain lakes were studied and compared with surveys conducted on the same lakes in 1993/2002 to assess biological responses to recent climate warming. Most lakes show significant shifts in this important group of aquatic insects towards assemblages typical of warmer conditions, while a subset exhibits stable or opposing trends driven by local catchment factors. These findings demonstrate that alpine aquatic insect communities are already responding to rising temperatures, with further ecological reorganization expected as warming continues.

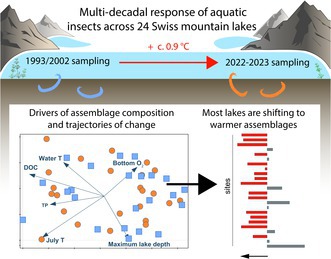

## 1 Introduction

Mountain lakes are important indicators of global change due to their high elevations, low water temperatures, and strong sensitivity to climatic and atmospheric forcings (Moser et al. 2019; Pastorino and Prearo 2020). Over the past 30 years, mean annual air temperatures in alpine regions have increased by ~0.9°C, well above the global average of ~0.5°C (Nigrelli and Chiarle 2023), and further warming of ~0.25°C–0.36°C per decade is projected for the following century in the European Alps (Gobiet et al. 2014). Within Switzerland, warming shows an elevation‐dependent signal, with particularly strong increases projected in mountainous regions of eastern Switzerland (Rottler et al. 2019; Fischer et al. 2022). Even small temperature fluctuations can strongly alter ice‐ and snow‐cover durations and surface water temperatures in mountain lakes (Weckström et al. 2016; Woolway et al. 2021) and are already driving ecological change (Oleksy et al. 2020; Vitasse et al. 2021). While mountain lakes are typically less affected by agriculture than lowland lakes (Moser et al. 2019; Rogora et al. 2020), they experience a range of anthropogenic pressures such as fish introduction, pasturing, tourism, and catchment disturbances (Frossard et al. 2023; Tiberti et al. 2025), which can reinforce or obscure climatic signals (Adrian et al. 2009). Ecosystem responses are also often nonlinear and may involve time lags (Adrian et al. 2009; Catalan et al. 2009). Consequently, the extent to which European mountain lakes and their biotic communities have responded to recent climatic warming remains uncertain, as large‐scale, multidecadal surveys of sensitive indicator species are scarce in most European mountain regions.

Lake sediments preserve biological indicators, making them valuable archives for palaeoecological reconstructions and for assessing ecosystem responses on decadal or longer time scales (Battarbee et al. 2009; Moser et al. 2019). Among these indicators, chironomids, aquatic insects within the Diptera (true flies), are well‐suited as ecological indicators because they are taxonomically diverse with species‐specific ecological niches. The sclerotized head capsules of larval chironomids are well preserved in lake sediments, allowing identification of subfossil remains at the genus, species, or morphotype level (Brooks et al. 2007). Moreover, they occupy a wide range of ecological conditions, and consequently, changes in their assemblages can capture environmental shifts within individual lake systems (Hofmann 1986). Assemblage distributions are strongly linked to summer temperatures (Marziali and Rossaro 2013; Foucault et al. 2018), forming the basis for widely used quantitative temperature inference models (e.g., Heiri et al. 2011; Suranyi et al. 2025). Moreover, their assemblages appear strongly driven by changes in water chemistry variables expected to change with increasing temperatures, such as dissolved organic carbon (DOC) and nutrient concentrations (Luoto 2011; Luoto et al. 2016). However, earlier Alpine studies that failed to detect clear chironomid assemblage responses to 20th‐century warming suggested that widespread salmonid introductions may have altered some lake ecosystems and influenced chironomid responses to recent warming (e.g., Heiri and Lotter 2003; Von Gunten et al. 2008). Longer‐term records further illustrate that human land use, including pasturing, have affected mountain lakes and shifted chironomid assemblages toward warmer‐type states (Heiri and Lotter 2003; Tarrats Sada et al. 2017). It therefore remains unclear whether recent land use changes, together with fish introductions, may modify or partly counteract the effects of ongoing climatic warming on chironomid assemblages.

In the Swiss Alps, multi‐lake studies of aquatic insect assemblages were conducted in 1993 and 2002 (e.g., Lotter et al. 1997, 1998; Bigler et al. 2006; Heiri et al. 2011). These campaigns aimed to assess the distribution of aquatic indicators relative to prevailing environmental conditions, and to provide a basis for interpreting downcore analyses of aquatic proxy indicators in lake‐sediment records. Because multi‐lake surveys of living aquatic insect communities in mountain lakes are logistically demanding and time‐consuming, these campaigns focused on chironomid larval remains in lake surface sediment samples, which typically integrate assemblage composition over several years. Although not originally intended for this purpose, these surveys now provide an extensive historical documentation of the distribution of chironomid larvae in a large number of Swiss mountain lakes ca. 25–30 years ago, which can be compared with their present distribution to study how chironomid community composition has reacted to the observed, recent warming trend in summer temperatures.

For this study, we revisited 24 lakes surveyed in 1993/2002 and collected new surface‐sediment samples following the same approach as earlier campaigns. Chironomid remains were analysed and compared with detailed assessments of water chemistry and limnological conditions to determine the present‐day relationship between chironomid assemblage composition and environmental variables. We then compared our results with the earlier chironomid survey data to assess how chironomid assemblage composition changed over the past decades. Given decades of regional warming in the Alps and the well‐established link between chironomid distributions and summer temperature, we hypothesize that chironomid assemblages will shift towards more warm‐adapted taxa and assemblages typical of more productive, less oxygenated conditions, since productivity and oxygen are closely correlated with elevation and temperature in the Swiss Alps. At the same time, we expect site‐specific responses of chironomid assemblages to recent warming. Moreover, secondary environmental variables, threshold effects, and delayed ecosystem responses may lead to muted or absent assemblage shifts despite ongoing warming. Specifically we intend to assess (i) the present distribution of chironomid assemblages with respect to environmental variables potentially determining aquatic insect distributions in Swiss mountain lakes, (ii) the extent and directionality of shifts in chironomid assemblage composition at these sites over the past 20–30 years and thereby (iii) whether our results can provide evidence for a widespread response in mountain lake conditions and ecosystem states to the pronounced climatic warming during recent decades.

## 2 Materials and Methods

### 2.1 Study Region

**FIGURE 1:**
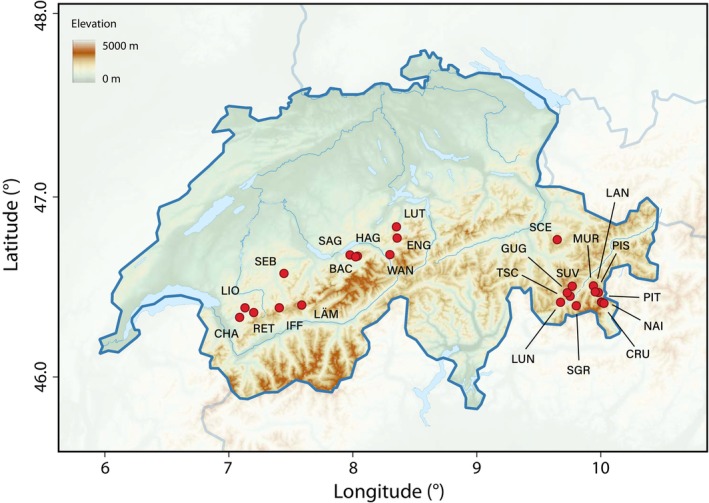
Map of Switzerland with the location of the 24 sampled lakes labelled with respective lake code. Map was created with QGIS (v. 3.44.5), and elevation data were obtained from the SwissALTIRegio digital height model (Federal Office of Topography swisstopo 2023).

A total of 24 mountain lakes were re‐sampled across the Northwestern and Eastern sectors of the Swiss Alps (Figure 1). The study sites are from the subalpine and alpine vegetation zone, spanning a wide range of thermal and precipitation regimes (Lotter et al. 1997; Bigler et al. 2006). Most lakes are situated above the tree line, with alpine meadows and rocky terrain dominating their catchments, whereas a few are located below the tree line. Clear‐water, oligo‐ to mesotrophic conditions typical of high‐elevation lakes characterize the study sites. Lake morphometry varies across sites, with differences in depth, catchment size and geometry (Table 1), although many sites show relatively shallow conditions (< 10 m). Lakes in the eastern Swiss Alps are mainly located in areas with igneous bedrock, primarily granite and gneiss, while in the northwestern Swiss Alps, sedimentary bedrock, mainly sandstone and limestone, is more common (Geological Atlas of Switzerland 1:25,000, Federal Office of Topography swisstopo 2026a).

**TABLE 1:** Major environmental variables of the investigated sites with surface and bottom‐water values shown for limnological measurements.

Lake name	Lake code	Sampling date DD.MM.YY	Coordinates		July Air T (°C)	Permafrost possibility (%)	Elevation (m a.s.l.)	Max. catchment elevation (m a.s.l.)	Catchment area (km^2^)	Max. depth (m)	Secchi depth (m)	Water T (°C)		Conductivity (μS @ 25C)		Oxygen (mg L^−1^)		pH	
			Latitude (N)	Longitude (E)															
												Top	Top	Top	Bot	Top	Bot	Top	Bot
Lej da Pischa Süd	PIS	12.09.23	46°28′17.14”N	9°58′44.54″ E	6.1	86.1	2770	3166	1.62	14.5	6.2	12.6	10.6	155	157	8.6	9.2	8.22	8.82
Lej Muragl	MUR	11.09.23	46°30′29.86”N	9°56′22.04″ E	6.3	89.6	2714	3199	0.52	6.3	6.3	12.3	10.7	162	170	9.1	9.7	8.07	8.03
Lej Sgrischus	SGR	15.08.23	46°23′51.90”N	9°48′5.23″ E	6.9	92.8	2618	3333	1.02	6.6	6.05	13.7	NA	130	NA	8.9	NA	8.76	NA
Lej da la Tscheppa	TSC	01.09.22	46°27′3.12”N	9°45′4.45″ E	6.8	57.5	2617	3075	0.66	27.4	10.7	13.4	8.6	52	51	7.9	9.1	8.20	7.73
Lej Suvretta	SUV	17.08.23	46°30′24.18”N	9°46′6.88″ E	6.9	62.2	2602	3143	0.45	8.4	2.25	11.5	10.0	145	154	8.9	9.4	8.29	8.43
Lej Languard	LAN	13.09.23	46°28′31.20”N	9°57′12.88″ E	7.2	93.8	2592	3050	0.55	9	NA	12.6	NA	191	NA	8.6	NA	7.78	NA
Lägh dal Lunghin	LUN	18.08.23	46°25′1.69”N	9°40′27.20″ E	7.7	44.3	2485	2932	1.18	20.6	2.25	12.7	5.0	254	565	8.7	2.6	8.34	7.30
Hagelseewli	HAG	17.08.22	46°40′23.38”N	8° 2′8.23″ E	7.8	38.7	2338	2622	0.29	18.9	6.7	12.2	4.8	158	250	9.1	3.2	8.64	7.71
Lagh da la Cruseta	CRU	03–04.08.22	46°24′41.70”N	10° 1′29.57″ E	9.7	12.5	2304	2483	0.14	11.2	7.6	18.3	16.9	111	111	7.8	7.9	8.25	8.30
Lämmerensee	LAM	08.08.23	46°24′6.44”N	7°35′13.34″ E	8.9	7.2	2296	2856	1.22	3.5	2.8	10.1	9.4	152	171	9.3	9.3	8.53	8.34
Güglia	GUG	02.09.22	46°28′12.53”N	9°43′40.61″ E	9.1	18.6	2270	2760	0.12	3.6	3.5	14.5	14.5	124	124	7.8	7.8	8.51	8.50
Bachalpsee	BAC	17–18.08.22	46°40′10.00”N	8° 1′24.00″ E	8.2	21.6	2266	2748	1.59	15.9	5.25	15.5	6.0	149	190	9.2	8.5	8.76	8.10
Lej Nair	NAI	03.08.22	46°24′59.08”N	10° 0′28.62″ E	10.2	15.5	2223	2895	1.82	11.6	7.85	16.7	16.1	49	49	7.6	7.5	7.82	7.59
Lej Pitschen	PIT	02.08.22	46°25′8.01”N	10° 0′13.98″ E	10.3	13.7	2217	2895	2.13	4.9	4.9	15.5	15.1	57	57	7.7	7.5	6.06	6.84
Wannisbortsee	WAN	22.08.23	46°40′54.37”N	8°17′51.96″ E	9.2	7.1	2104	2924	1.21	14.6	7.8	12.7	9.7	52	43	9.4	10	7.83	8.39
Iffigsee	IFF	13.09.22	46°23′12.70”N	7°24′20.07″ E	10.1	29.6	2065	2937	4.33	31.5	3.05	12.0	4.4	153	183	8.9	7.9	8.59	8.33
Sägistalsee	SAG	16.08.22	46°40′48.39”N	7°58′34.50″ E	10.4	7.7	1936	2571	4.87	9.1	9.1	17.2	12.4	261	353	8.4	8.6	8.14	7.62
Schwellisee	SCE	02.08.23	46°45′52.84”N	9°38′51.71″ E	11.5	35	1930	2980	9.82	11.6	11.6	10.4	10.0	275	276	10	10	8.48	8.57
Engstlensee	ENG	23.08.23	46°46′26.06”N	8°21′22.87″ E	10.7	17.2	1850	3041	7.47	50.8	11.5	17.7	4.6	173	213	8.3	3.2	8.30	7.77
Lac Lioson	LIO	22.09.23	46°23′10.94”N	7° 7′44.82″ E	11.1	0.7	1847	2477	1.43	28.5	11.8	14.2	4.6	164	202	9.5	0.1	8.57	7.39
Seebergsee	SEB	04.08.23	46°34′39.11”N	7°26′36.19″ E	11.3	0	1831	2058	0.23	15.5	2.8	14.4	4.6	179	301	9.9	0	8.94	7.35
Lutersee	LUT	11.08.23	46°50′8.49”N	8°21′3.19″ E	11.6	0	1703	2245	0.58	4.9	2.05	13.5	11.3	135	150	11	9.3	8.74	7.99
Lac des Chavonnes	CHA	10.08.22	46°20′0.04”N	7° 5′7.51″ E	12.1	0	1688	1993	0.66	25.7	5.3	20.0	4.1	172	227	8.4	0	8.62	7.59
Lac Retaud	RET	18.09.23	46°21′36.36”N	7°11′58.16″ E	12.1	0	1685	2170	0.23	4.5	1.65	17.3	17.2	282	288	8.5	6.7	8.23	7.90

Lake name	Lake code	DOC (mg L^−1^)		TOC (mg L^−1^)		TN (mg L^−1^)		TP (mg L^−1^)		PO_4_ (mg L^−1^)		Si (mg L^−1^)		SAC‐254 (1 m^−1^)		SAC‐436 (1 m^−1^)		Carbonates (mmol L^−1^)		Alkalinity (mmol L^−1^ pH 4.5)	
		Top	Bot	Top	Bot	Top	Bot	Top	Bot	Top	Bot	Top	Bot	Top	Bot	Top	Bot	Top	Bot	Top	Bot
Lej da Pischa Süd	PIS	0.41	0.45	0.63	0.57	0.09	0.09	0.0030	0.0045	0.0092	0.0138	0.97	0.91	0.446	0.472	0.036	0.044	0.406	0.413	0.39	0.39
Lej Muragl	MUR	0.30	0.30	0.47	0.42	0.23	0.24	0.0030	0.0036	0.0092	0.0110	0.97	0.98	0.430	0.417	0.035	0.043	0.427	0.438	0.40	0.42
Lej Sgrischus	SGR	0.70	0.63	0.93	0.82	0.16	0.16	0.0036	< 0.0030	0.0110	< 0.0092	0.77	0.77	0.467	0.757	0.126	0.194	0.801	0.801	0.78	0.78
Lej da la Tscheppa	TSC	0.47	0.50	0.59	0.78	0.15	0.12	< 0.0030	0.0103	< 0.0092	0.0316	0.19	0.38	0.35	0.60	0.039	0.078	0.424	0.420	0.40	0.40
Lej Suvretta	SUV	0.63	0.58	0.89	0.88	0.09	0.09	0.0050	0.0074	0.0153	0.0227	0.60	0.51	0.957	0.862	0.049	0.048	0.712	0.744	0.69	0.72
Lej Languard	LAN	0.54	0.49	0.71	0.73	0.26	0.27	0.0085	0.0176	0.0261	0.0540	0.74	0.75	0.508	0.592	0.050	0.049	0.541	0.545	0.52	0.52
Lägh dal Lunghin	LUN	0.49	0.33	0.8	0.44	0.12	0.11	< 0.0030	0.0106	< 0.0092	0.0325	0.94	2.90	0.556	0.515	0.030	< 0.030	0.776	1.180	0.74	1.15
Hagelseewli	HAG	0.47	0.49	0.59	1.00	0.14	0.18	0.0082	0.0315	0.0251	0.0966	0.52	2.20	0.53	0.97	0.064	0.085	1.410	2.000	1.39	1.98
Lagh da la Cruseta	CRU	1.00	1.10	1.20	1.10	0.13	0.12	0.0061	0.0114	0.0187	0.0350	0.43	0.46	1.630	1.510	0.084	0.074	0.491	0.488	0.47	0.47
Lämmerensee	LAM	0.83	0.80	1.2	1.2	0.10	0.09	0.00	< 0.0030	0.0107	< 0.0092	0.23	0.24	0.922	0.914	0.063	0.087	1.550	1.560	1.53	1.54
Güglia	GUG	2.30	2.20	2.70	2.70	0.25	0.25	0.0037	0.0040	0.0113	0.0123	1.60	1.60	1.43	1.32	0.085	0.065	0.723	0.708	0.70	0.68
Bachalpsee	BAC	0.83	0.56	1.20	0.87	0.11	0.11	0.0043	0.0082	0.0132	0.0251	0.42	0.68	1.04	1.04	0.062	0.065	1.500	1.890	1.48	1.86
Lej Nair	NAI	1.40	1.40	1.50	1.60	0.14	0.16	0.0054	0.0206	0.0166	0.0632	0.89	0.86	2.980	2.970	0.139	0.157	0.278	0.281	0.26	0.26
Lej Pitschen	PIT	2.00	1.80	2.40	2.10	0.31	0.33	0.0119	0.0182	0.0365	0.0558	0.86	0.86	4.210	4.070	0.238	0.235	0.320	0.320	0.30	0.30
Wannisbortsee	WAN	0.27	0.37	0.38	0.45	0.17	0.14	< 0.0030	0.0046	< 0.0092	0.0141	0.94	0.87	0.470	0.673	< 0.030	0.043	0.288	0.271	0.27	0.25
Iffigsee	IFF	0.40	< 0.2	0.57	0.37	0.20	0.23	< 0.0030	0.0032	< 0.0092	0.0098	0.23	0.85	0.41	0.48	0.032	0.045	1.480	1.750	1.46	1.72
Sägistalsee	SAG	0.76	0.45	0.89	0.62	0.11	0.10	< 0.0030	0.0059	< 0.0092	0.0181	0.59	0.78	1.03	0.79	0.044	< 0.030	2.700	3.500	2.68	3.47
Schwellisee	SCE	0.46	0.34	0.54	0.42	0.16	0.15	< 0.0030	< 0.0030	< 0.0092	< 0.0092	1.40	1.30	0.526	0.540	< 0.030	0.044	2.060	2.060	2.03	2.04
Engstlensee	ENG	0.37	0.22	0.53	0.41	0.17	0.17	< 0.0030	0.0043	< 0.0092	0.0132	0.58	1.70	0.385	0.420	< 0.030	< 0.030	1.560	1.980	1.53	1.96
Lac Lioson	LIO	0.88	0.44	1.10	0.75	0.11	0.38	< 0.0030	0.0165	< 0.0092	0.0506	0.23	1.70	1.03	1.07	0.055	0.130	1.690	2.080	1.67	2.05
Seebergsee	SEB	3.10	1.20	3.6	2.2	0.18	1.20	0.01	0.19	0.0273	0.5890	0.28	2.70	2.490	2.160	0.134	0.134	1.840	2.790	1.82	2.77
Lutersee	LUT	2.00	1.90	2.3	2.5	0.20	0.26	0.02	0.03	0.0518	0.0788	0.45	0.51	5.120	5.040	0.381	0.387	1.450	1.510	1.43	1.49
Lac des Chavonnes	CHA	1.50	1.40	1.60	3.60	0.26	1.40	0.0063	0.0623	0.0193	0.1910	0.13	2.70	2.51	3.92	0.077	0.199	1.770	2.240	1.75	2.21
Lac Retaud	RET	2.40	2.40	3.40	3.60	0.28	0.29	0.0148	0.0221	0.0454	0.0678	1.30	1.40	4.68	4.78	0.420	0.485	2.750	2.780	2.72	2.74

### 2.2 Fieldwork and Environmental Data

Fieldwork at each lake was conducted on a single day during the main growing season and stratification period, between 2nd of August and 22nd of September in 2022–2023. Short sediment cores were taken with an 86 mm inner diameter Pylonex HTH corer (Renberg and Hansson 2008) from the deepest section of each lake. The top 1.5 cm of sediment was sampled in 0.5 cm intervals in the field and stored in plastic bags in a cold room (4°C) until further processing. Based on previously reported sedimentation rates, surface sediment samples collected in our study are expected to encompass between ~3 and 9 years (Lotter et al. 2000; Heiri and Lotter 2003; Lotter et al. 2006; Von Gunten et al. 2008; Schwörer et al. 2014). Probes for dissolved oxygen (WTW FDO 925), pH (WTW SensoLyt 900‐P), conductivity, and temperature (WTW TetraCon 925) were used to measure depth profiles, with measurements at 1‐m intervals at each lake. For sites LAN and SGR, only surface water measurements of temperature, oxygen, pH, and conductivity were retrieved due to unfavourable and potentially hazardous weather conditions that limited time at the study sites.

Four 250 mL water samples were collected from each lake using a Niskin bottle (two at 1 m water depth and two 1 m above the sediment) and stored in glass bottles at 4°C until further analyses. Water chemistry analyses carried out at the Amt für Umwelt und Energie of the Canton of Basel‐Stadt included alkalinity, DOC, total organic carbon concentration (TOC), UV and visible absorption of DOC, expressed as spectral absorption coefficient at 254 and 436 nm (SAC‐254 and SAC‐436), total nitrogen concentration (TN), total phosphorus concentration (TP), silica concentration, and carbonate concentration (see Table S1 for details). Detection limits for DOC, TOC, TN, and TP were 0.2, 0.25, 0.05, and 0.003 mg L^−1^, respectively. Mean July air temperatures were derived from climate normals for 1991 to 2020 and estimated using linear regression‐based spatial interpolation applied to 1 km grid datasets from the Swiss Federal Office of Meteorology and Climatology (MeteoSwiss).

Catchment area size, maximum catchment elevation, and fraction of the lake catchment possibly affected by permafrost (permafrost possibility) were calculated in ArcGIS Pro (v.3.3.0) using the SwissALTI3D digital elevation model (Federal Office of Topography swisstopo 2024) and the permafrost distribution in Switzerland dataset provided by the Swiss Federal Office for the Environment (Federal Office of Topography swisstopo 2026b).

### 2.3 Chironomid Analysis

Analysis of chironomid remains in surface sediment samples followed standard procedures (e.g., Walker et al. 1991; Brooks et al. 2007). Subsamples of freeze‐dried sediment (ca. 0.01–5 g) were deflocculated at 70°C in KOH (10%) for 30 min and sieved through a 100 μm mesh‐sized sieve. Chironomid head capsules and other invertebrate remains were sorted from the sieved residue using a Bogorov tray (Gannon 1971) under a stereomicroscope (20–50× magnification) and mounted in Euparal. We used a minimum target of 50 head capsules, commonly recommended as threshold for numerical analysis of subfossil chironomid remains (Heiri and Lotter 2001; Larocque 2001; Quinlan and Smol 2001). Depending on the number of chironomid remains, the topmost 0.5, 1.0, or the entire 2 cm interval was analysed. Head capsules were identified to genus or species morphotype under a compound microscope (100–400× magnification), following Wiederholm (1983), Kowalyk (1985), Schmid (1993), Rieradevall and Brooks (2001), and Brooks et al. (2007). Head capsules with > 50% of the mentum intact were counted as one individual, half as 0.5 individuals, and < 50% as 0 individuals (e.g., Bolland et al. 2022). Specimens not identified to the highest taxonomic resolution (e.g., due to missing structures such as mandibles) were assigned to categories based on the ratio of identified specimens within the same sample (Bolland et al. 2020; Heiri and Engels 2026).

Chironomid analysis of surface sediment samples collected in 1993/2002 followed procedures similar to those applied to the new samples and were previously described by Lotter et al. (1997) and Bigler et al. (2006). Lotter et al. (1997) analysed dry sediment, whereas the study of Bigler et al. (2006) used wet sediment, with slight differences in the KOH treatment, which are not expected to influence the results. Slides of the study by Lotter et al. (1997) and Bigler et al. (2006) were re‐identified, and taxonomic resolution adjusted to the taxonomic categories described in Brooks et al. (2007) in an earlier study (Heiri and Lotter 2010). To combine this dataset with the newly developed chironomid assemblage data from the Swiss Alps, minor taxonomic adjustments were necessary. Several morphotypes were merged at the genus level (e.g., *Diamesa steinboecki* and *Diamesa zernyi/cinerella*‐type merged to *Diamesa*), when genus‐level identification was achieved in only one of the datasets. Various morphotypes of the genera *Cricotopus* and *Orthocladius* were merged for consistency. Taxa identified only to subfamily‐ or tribe‐level were excluded. Older samples (1993/2002) were marked with site names followed by “2” to distinguish them from modern samples. The complete list of sites and abbreviations is provided in Table 1.

### 2.4 Numerical Analysis

To analyse trends in taxon richness across samples with varying sample sizes at a constant sample count, rarefaction analysis was performed in PAST 5.2 (Hammer and Harper 2001). Further analyses of chironomid data were based on percentage data. For numerical analyses, average water‐column values of temperature, oxygen, pH, and conductivity were used, with surface (1 m below water surface) and bottom water (1 m above sediment surface) measurements examined for parameters that exhibited large changes throughout the water column. For water chemistry parameters that were only measured on surface and bottom waters (DOC, TOC, TN, TP, phosphate (PO_4_), silica, SAC‐254 and SAC‐436, carbonates, and alkalinity), a weighted averaging (WA) procedure as described in Lotter et al. (1998) and Bigler et al. (2006) was employed to provide estimates of mean values across the water column. Because water column measurements for temperature, oxygen, pH, and conductivity were missing from LAN and SGR, except for surface measurements, these values were replaced by the mean for the variable from lakes with similar depth (±6 m), elevation (±400 m), and surface values (±3.5°C, 0.5 mg L^−1^, 0.8 and 80 μS for temperature, oxygen, pH, and conductivity, respectively) for each parameter. Values below detection limit were replaced with the midpoint between zero and the detection limit. Variables with clearly right‐skewed distributions in histograms were log‐transformed. These included maximum water depth, DOC, TOC, TN, TP, PO_4_, dissolved silica, SAC‐254, SAC‐436, carbonates, and alkalinity. Newly collected water chemistry data in this study were initially analysed in CANOCO 5.12 (Šmilauer and Lepš 2014) using Principal Components Analysis (PCA) on a correlation matrix to explore and identify the dominant environmental gradients across lakes and to assess co‐variability among variables. Chironomid assemblage data were initially explored with detrended correspondence analysis (DCA; Hill and Gauch Jr 1980) using detrending‐by‐segments, square‐root transformation of species percentage data, and downweighing of rare taxa, to determine the lengths of the compositional gradients and estimate whether linear or unimodal species response models were most appropriate (ter Braak and Prentice 1988). All subsequent numerical analyses of the chironomid datasets [canonical correspondence analysis (CCA) and detrended CCA (DCCA)] were conducted using unimodal species‐response models, as the dataset exhibited compositional gradient lengths exceeding 2 standard deviation (SD) units, suggesting that unimodal numerical techniques are appropriate (Hill and Gauch Jr 1980). All following ordinations based on the chironomid assemblage data were based on square‐root transformed percentages with down‐weighting of rare taxa.

A series of DCCAs was first conducted to assess the strength of the relationship between chironomid assemblage data from the newly sampled lakes and the observed environmental variables. Statistical significance was assessed by Monte Carlo permutation tests (9999 permutations, ter Braak 1990). In a second step, we conducted a DCCA using a subset of variables selected for their statistical significance, strong relationships with the chironomid assemblage data, and ecologically meaningful roles based on earlier surveys of chironomid species‐environment relationships and/or their representation of different major environmental gradients in the dataset. This DCCA was used to visualize species‐environment relationships together with the correlation structure (for environmental variables) in our newly collected dataset. Samples collected in the earlier campaigns in 1993/2002 were passively plotted to assess trajectories of change, and the extent to which chironomid assemblage composition shifted toward assemblage states typical of different environments over the past decades. Interpretations focused on biplots showing weighted average (WA) scores, since these primarily reflect the position of sites in DCCAs as determined by their community compositions (Oksanen 2016). Finally, we examined the extent to which assemblage composition changed along DCCA axis 1 between samples from the campaign in 1993/2002 and our new survey in 2022–2023, based on the DCCA with selected environmental variables, as well as DCCAs with single environmental variables. This served to assess whether there was evidence for a significant overall shift towards DCCA axis 1 values typical for environmental conditions expected to be prevalent in warmer lakes, such as higher overall water temperatures and higher DOC that typically occur at sites with higher July air temperatures. Overall significance of DCCA axis 1 changes in our dataset was assessed with the function wilcox.test in RStudio (v.01.0 + 392) using a one‐sided non‐parametric paired test (Wilcoxon signed‐rank test) based on the differences in DCCA axis 1 scores of the newly collected samples versus the samples collected in 1993/2002. Non‐parametric Spearman's rank correlation was performed between these differences in the multi‐variable DCCA and elevation, maximum catchment elevation, and the proportion of the catchment potentially affected by permafrost, with the function cor.test in RStudio. Correlations were calculated for the entire dataset but also for the extreme 66% shifts in DCCA values (ignoring the 33% sites with low amplitude changes) to compare how correlations change if only sites with distinct variations in chironomid assemblage compositions were examined.

## 3 Results

### 3.1 Environmental Data

**FIGURE 2:**
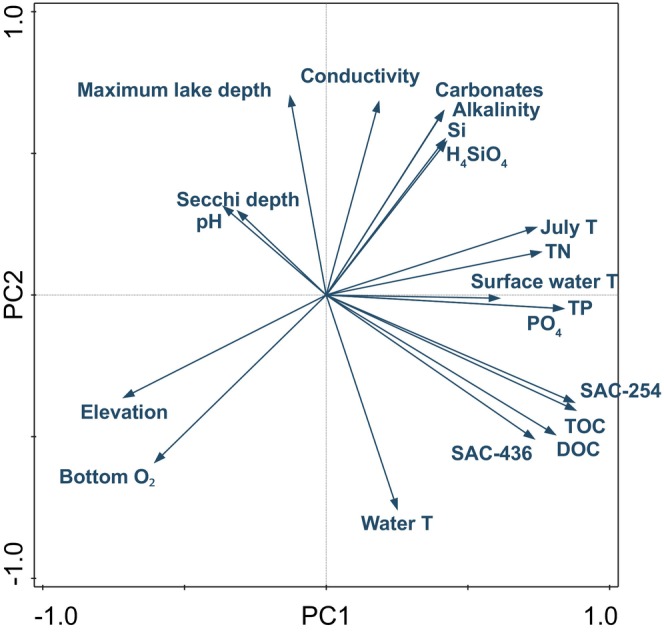
PCA scatterplot showing axis 1 and 2, illustrating the relationships between environmental variables (arrows) in the study sites.

The studied lakes predominantly exhibit cold, oligo‐ and mesotrophic conditions (Table 1), typical for mountain regions (e.g., Bigler et al. 2006). Lakes in the Engadine region of eastern Switzerland (e.g., PIS, MUR, and SUV; Figure 1) differ notably from those in the western region, with lower carbonate content, alkalinity, and conductivity. These differences partly reflect underlying catchment geology, with crystalline bedrock being more prevalent in the Engadine, compared to more calcareous bedrock in the western Alps. Several water chemistry parameters show relationships with elevation, which in the Alps is closely linked to (summer) temperature (Lotter et al. 1997; Schär et al. 1998). TOC and DOC of the water tend to be highest in lower‐elevation lakes (e.g., SEB, RET, and CHA) and are correlated positively with water temperature and nutrient concentration (e.g., TN and TP; Figure 2), consistent with patterns previously documented in the Alps (e.g., Lotter et al. 1997, Müller et al. 1998). At most sites, chemical variables showed only minor differences between surface and bottom waters. Exceptions are SEB and CHA, which exhibited pronounced variation in several variables, including lake water temperature, oxygen, and nutrient concentrations. These two lakes, among the few with elevated nutrient concentrations in the dataset, are also sufficiently deep to develop strong thermal stratification (thermocline at 6 m for CHA and 7 m for SEB), resulting in anoxic bottom waters at the time of coring. LIO also exhibits anoxic conditions in the hypolimnion, although differences in nutrient concentrations are less pronounced than observed in CHA and SEB. Other lakes in the dataset similarly showed a distinct thermal stratification (e.g., HAG, IFF, LUN, and ENG) at the time of coring. However, most of these lakes exhibit low nutrient concentrations in both the epilimnion and hypolimnion.

### 3.2 Chironomid Analysis

**FIGURE 3:**
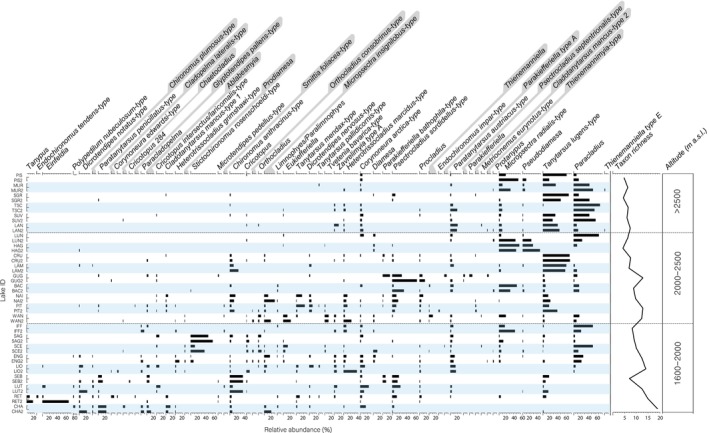
Distribution of the most abundant chironomid taxa in the investigated surface sediment samples ordered according to altitude. Lake codes followed by the number 2 indicate surface sediment samples collected 1993/2002 (Heiri and Lotter 2010), while lake codes without numbers indicate samples collected in the course of this study.

A total of 57 chironomid taxa were found in the surficial sediments across the 24 investigated lakes. Rarefaction analysis indicated that taxon richness tends to increase with decreasing elevation (Figure 3). Most sites at these lower elevations are located in the northwestern Alps, below the treeline, and generally exhibit higher nutrient concentrations and organic matter content, as well as elevated surface‐water temperatures. These lakes contain a higher abundance of taxa whose larvae may occur in meso‐ to eutrophic lakes (e.g., *Cladopelma*, *Polypedilum* and *Chironomus plumosus* ‐type; Figure 3). Rarefied richness decreases with increasing elevation, and at elevations between 2000 and 2500 m a.s.l., many of the dominant taxa are present in all investigated altitudinal zones, e.g., *Psectrocladius sordidellus* ‐type, *Corynoneura arctica* ‐type, *Procladius*, *Paratanytarsus austriacus*‐type and *Heterotrissocladius marcidus* ‐type. Lakes at elevations above 2500 m a.s.l. contain the lowest rarefied richness and are dominated by cold oligotrophic assemblages with *Tanytarsus lugens*‐type, *Micropsectra radialis*‐type, *Pseudodiamesa* and *Paracladius* being the dominating taxa.

When chironomid assemblages in the newly obtained samples are compared with data from 1993/2002 (Figure 3), it becomes apparent that the overall distribution of chironomid taxa relative to elevation remains comparable across the two sampling campaigns, with e.g., *Cladopelma*, *Polypedilum*, and *C. plumosus* ‐type, again mainly found in the same lower elevation lakes, and *M. radialis* ‐type, *Pseudodiamesa*, and *Paracladius* mainly found in high elevation sites. However, distinct changes are apparent at some sites. For example, pronounced decreases in *P. sordidellus* ‐type are apparent at GUG and BAC between the 1993/2002 sampling and our 2022–2023 campaign. *M. radialis* ‐type decreases in IFF, MUR, and PIS across this interval, whereas a moderate increase is observed for this taxon in BAC. Other taxa that show noticeable changes at some sites include, for example, *Stictochironomus rosenschoeldi* ‐type, *H. marcidus* ‐type, *Pseudodiamesa*, or *T. lugens* ‐type. At several sites, taxa were found in the 2022–2023 campaign that had not yet been observed in the 1993/2002 campaign, such as *Polypedilum nubeculosum* ‐type in CHA, RET, LUT, and ENG, and *Chironomus anthracinus* ‐type in SGR, TSC, and SUV. The opposite pattern, with taxa found in 1993/2002 no longer detected in 2022–2023, was also observed for other sites, such as *Cladopelma lateralis*‐type at GUG or *Ablabesmyia* at CRU.

### 3.3 Numerical Analysis

PCA revealed strong correlations among several variables (Figure 2) and identified redundant variables that could be omitted or treated as representing the same environmental gradient for subsequent ordination analysis. For example, DOC, TOC, SAC‐436, and SAC‐254 were highly positively correlated and represent the gradient of dissolved and particulate organic carbon in our lakes. Similarly, PO_4_, TP, and TN were positively related, reflecting co‐varying nutrient concentrations, whereas water temperature and July air temperature were positively correlated with each other and negatively correlated with elevation. Strong positive correlations were also apparent between dissolved silicic acid (H_4_SiO_4_) and dissolved silica (Si), and between conductivity, carbonate content, and alkalinity.

DCCA was used to quantify relationships between chironomid assemblages in our newly collected lake‐surface sediment samples and environmental variables assessed across the surveyed lakes (Table 2). Variables independently explaining the largest amount of variance include TOC, DOC, SAC‐254, SAC‐436, July air temperature, oxygen, TN, elevation, TP, PO_4_, and surface water temperature; all of these were identified as statistically significant during analysis.

**TABLE 2:** Percentage variance of chironomid assemblages in the study lakes explained by selected environmental variables when considered individually in DCCA. Bold indicates a *p*‐value of < 0.05.

	Simple	*p*
TOC	**15.9**	0.0001
DOC	**15.4**	0.0001
SAC‐254	**15.2**	0.0001
SAC‐436	**13.2**	0.0001
July Air T	**12.8**	0.0002
O_2_	**12.2**	0.0002
TN	**11.4**	0.0006
Elevation	**11.4**	0.0005
TP	**10.4**	0.0017
PO_4_	**10.4**	0.0012
Surface water T	**9.9**	0.0024
Water T	**9**	0.0052
Bottom O_2_	**7.8**	0.0191
pH	7.5	0.0616
Maximum lake depth	7.1	0.0883
Carbonates	6	0.1013
Alkalinity	6	0.105
Secchi depth	5.7	0.1377
Si	5.4	0.1935
H_4_SiO_4_	5.4	0.1923
Conductivity	5.2	0.2359

**FIGURE 4:**
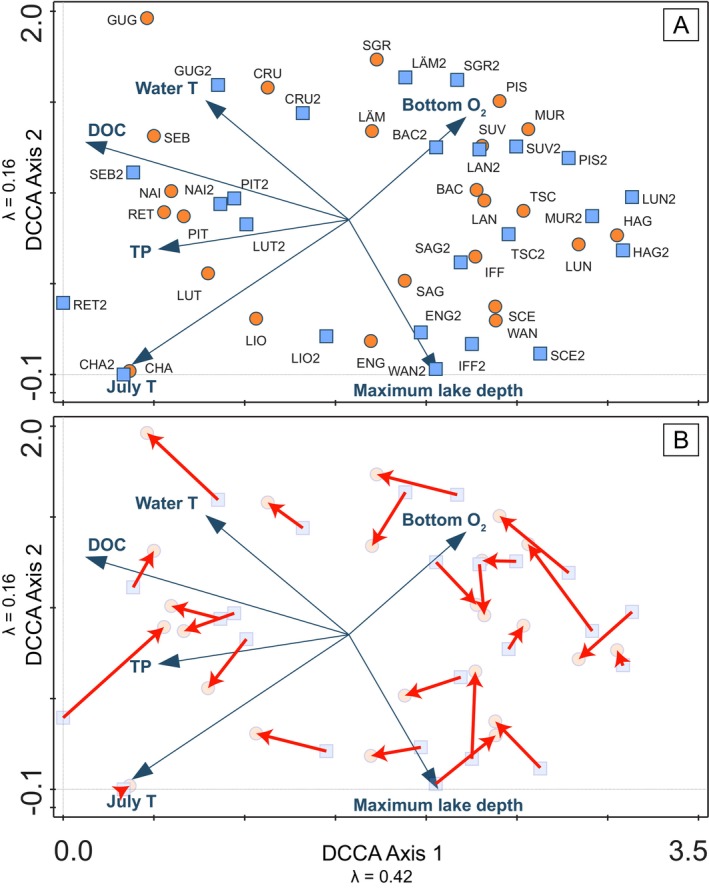
(A) DCCA biplots showing axis 1 and 2 of a DCCA of chironomid assemblage data constrained by select environmental variables. The DCCA was calculated based on our new survey data (2022–2023), with the results from earlier campaigns in 1993/2022 plotted passively. WA scores of active (2022–2023; orange) and passive (1993/2002; blue) samples are shown. (B) Red arrows indicate direction of change from older samples (blue) to more recent (orange) samples.

To visualize species‐environment relationships and to assess trajectories of change between 1993/2002 and 2022–2023, we calculated a DCCA based on selected environmental variables. These variables were chosen based on their significant explanatory power in the chironomid assemblage data and/or were identified as important gradients of chironomid assemblage change in earlier studies. For strongly co‐varying variables (as apparent in the PCA results), only one representative variable was selected (Figure 2). Selected variables included DOC, July air temperature, TP, water temperature, maximum lake depth, and bottom oxygen concentration (Figure 4). Variables such as TN and PO_4_ individually explained significant proportions of variance but were excluded due to strong correlations with TP, whereas TOC, SAC‐254 and SAC‐436 were not selected due to their strong correlation with DOC. The first axis of the resulting DCCA (λ_1_ = 0.42) primarily separates the modern samples along the DOC gradient. Lakes with chironomid assemblages typical of higher DOC values are characterized by low DCCA axis 1 values (e.g., SEB, RET, and CHA), and assemblages more commonly found in lakes with lower DOC values by high DCCA axis 1 values (e.g., HAG, LUN, and MUR). Sites with low DCCA axis 1 values primarily consist of lakes at lower elevation with higher temperatures and nutrient concentrations (Figures 3 and 4), whereas sites with high values mainly represent more oligotrophic lakes at higher elevations. The second axis (λ_2_ = 0.16) is substantially weaker, and mainly appears to be associated with lake depth and, to some extent, water temperature and bottom oxygen.

**FIGURE 5:**
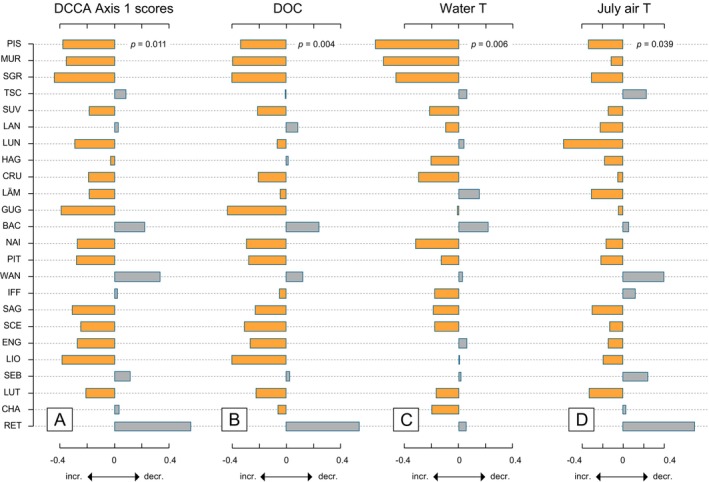
Differences in DCCA axis 1 values (ΔDCCA) between chironomid samples collected in this study (2022–2023) and samples collected in earlier campaigns in 1993/2002: (A) ΔDCCA of the analysis including DOC, July air temperature, bottom oxygen, TP, water temperature, and maximum lake depth shown in Figure 4; (B–D) ΔDCCA axis 1 scores of single‐variable DCCAs, including DOC, water temperature, and July air temperature. For A–D, negative ΔDCCA indicates that the modern assemblage is associated with negative DCCA axis 1 values and increasing values of DOC, water temperature, July air temperature and TP, as shown by the arrows at the bottom of the plots (decr. = decreasing, incr. = increasing). *p*‐values for a one‐sided Wilcoxon signed‐rank test are indicated for each plot.

Trajectories of change between axis 1 scores of modern and older samples from the same locality (Figures 4B and 5A) indicate that chironomid assemblages at most sites show a shift toward assemblage states associated with lower axis 1 values in the multi‐variable DCCA, conditions which, based on this analysis, coincide with higher DOC values, nutrient conditions, and temperatures. A few sites (TSC, LAN, IFF, SEB, CHA in Figure 4B) show minor shifts toward assemblage compositions typical of higher DCCA axis 1 scores associated with low‐DOC and low‐temperature sites. Furthermore, two sites (RET and WAN) show a strong trend toward assemblages typically found in lakes with higher DCCA axis 1 scores. Similar patterns are observed when DOC, water temperature, and July air temperature are included as single explanatory variables in DCCAs of our assemblage data (Figure 5B–D). In these analyses, most sites again show shifts towards assemblage states associated with higher DOC concentrations (Figure 5B), water temperatures (Figure 5C), and July air temperatures (Figure 5D). A Wilcoxon signed‐rank test confirms that this shift towards conditions typically found in warmer, lower elevation sites is statistically significant across the 24 lakes (*p* = 0.011) for changes in DCCA axis 1 of the analysis based on several environmental variables (Figure 5A), as well as for DOC (*p* = 0.004), water temperature (*p* = 0.006), or July air temperature (*p* = 0.039). However, individual lakes (e.g., BAC for DOC or LÄM for water temperature) exhibit moderate to large changes in assemblage composition in the opposite direction for all of these analyses (Figure 5B–D), similarly as observed in multi‐variable DCCA (Figure 5A).

**FIGURE 6:**
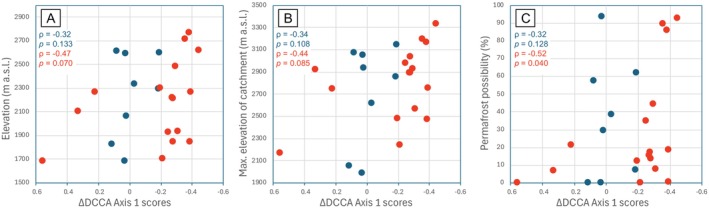
Differences in DCCA Axis 1 values (ΔDCCA) between chironomid samples collected in this study (2022–2023) and samples collected in earlier campaigns in 1993/2002 plotted versus elevation (A), maximum catchment elevation (B) and the proportion of the lake catchment potentially affected by permafrost (C) based on the analysis including DOC, July air temperature, bottom oxygen, TP, water temperature, and maximum lake depth shown in Figure 4. Red samples represent the extreme 66% of ΔDCCA values. Spearman rank correlation and significance values are provided for all (blue) and the 66% extreme samples (red).

A visual examination of our results suggests that changes towards DCCA axis 1 values typical for warmer, lower elevation conditions in the DCCA based on multiple environmental variables may be more pronounced and consistent in higher‐elevation lakes, whereas lower‐elevation sites show more inconsistent and variable responses (Figure 5). Spearman's rank correlation indicates only moderately strong, non‐significant relationships between DCCA axis 1 scores and elevation (ρ = 0.32, *p* = 0.133; Figure 6). The correlation was similarly strong with other catchment variables that potentially affect the hydrology of mountain lakes, such as maximum catchment elevation (ρ = 0.34, *p* = 0.108) or the fraction of the catchment potentially affected by permafrost (ρ = 0.32, *p* = 0.128). We also examined correlations including only sites with more pronounced DCCA axis 1 changes (Figure 5), to assess whether relations of chironomid assemblage change with these variables were more pronounced when only localities with clear evidence for shifting chironomid populations were examined. When only these localities were included, the strength of the correlations increased (ρ = 0.44–0.52), although the relationships remained not or only marginally significant (*p* = 0.040–0.085) (Figure 6).

## 4 Discussion

### 4.1 Environmental Drivers of Chironomid Distribution

Our assessment of the relationship between chironomid assemblage composition and environmental variables measured during this campaign supports and reinforces the findings of earlier studies on potential drivers of chironomid assemblage change in the study region. Single‐variable DCCAs identified variables representing organic carbon and dissolved organic matter (DOC, TOC, SAC‐254, SAC‐436), temperature (July air temperature, water temperature), nutrient concentrations (TN, TP, PO_4_), elevation, oxygen availability, and water depth as accounting for the highest statistically significant variability in the dataset when considered individually (Table 2).

Variables representing organic carbon and dissolved organic matter account for most variance in chironomid assemblage composition (TOC 15.9%, DOC 15.4%, SAC‐254 15.2%, and SAC‐436 13.2%). TOC is the strongest predictor in our dataset; however, DOC is more commonly used than TOC to explain variation in modern chironomid assemblages, as it reflects water‐column processes that influence larval habitat (Bigler et al. 2006; Larocque et al. 2006; Luoto et al. 2016). For this reason, and because DOC, TOC, and spectral absorption strongly covary in our dataset, the following discussion focuses on DOC, while acknowledging that TOC and other organic‐matter variables contribute to the observed pattern.

Previous studies have also identified DOC or TOC as significant explanatory variables for the distribution of chironomid assemblages (Larocque et al. 2001; Nyman et al. 2005; Bigler et al. 2006; Luoto et al. 2016), and in alpine regions, DOC is closely related to temperature (Heiri and Lotter 2008; Eggermont and Heiri 2012). DOC may support microbial production in freshwater systems and thereby indirectly serve as a carbon source for benthic macroinvertebrates (Demars et al. 2021). Furthermore, DOC can be expected to reflect, to some extent, the overall productivity of lake ecosystems and their catchments. DOC concentrations in the investigated lakes were low, averaging 1.1 ± 0.8 mg L^−1^ (SD). Bigler et al. (2006) argue that the strong relationship between DOC and chironomid assemblages at some sites in the Engadine area of southeastern Switzerland may be attributed to the strong influence of temperature on the studied lakes, rather than to DOC concentrations themselves. Although DOC concentrations are low, a clear separation between sites with lower and higher concentrations is apparent, with some lakes, such as RET, GUG, SEB, LUT, PIT, CHA, and NAI showing higher values (Table 1). Modest differences in DOC concentrations may influence key parameters in lake ecosystems affecting chironomid assemblages, such as light penetration (Forsström et al. 2013), as evidenced by the elevated SAC‐254 and SAC‐436 values at these sites (Table 1), and consequently, can influence oxygen availability at the lake bottom available for the larvae. Several variables representing nutrient concentrations in our study lakes, such as TN, TP, and PO_4_, also explain statistically significant variability in the dataset (Table 2) and are correlated with DOC (Figure 2). Between‐lake variations in productivity and nutrient availability have repeatedly been identified as possible drivers for changes in chironomid assemblage composition (Lotter et al. 1998; Brodersen and Lindegaard 1999; Bigler et al. 2006). Particularly, the availability of phosphorus, the limiting element for plant growth in many Swiss lakes (Schindler 1977; Lepori et al. 2022), has previously been suggested to influence lake productivity, trophic state, and indirectly also the food and oxygen availability for chironomid larvae in lakes (Wilson and Gajewski 2004; Bigler et al. 2006). Many of the sites with elevated DOC exhibit elevated nutrient concentrations and generally show lower dissolved oxygen concentrations (e.g., RET, CHA, LUT, SEB, PIT; Table 1), further illustrating a separation of these lakes by their physicochemistry. These combined characteristics may alter food‐web interactions and water‐column transparency, thereby influencing chironomid assemblages differently than at sites with lower DOC and nutrient levels.

As in many earlier surveys of chironomid distributions across multiple lakes, air temperature, elevation, and water temperature also show strong, statistically significant explanatory power in the dataset (Table 2). These variables are strongly intercorrelated and exhibit robust associations with DOC and nutrient concentrations in our dataset. Deepwater oxygen concentration was also identified as a significant explanatory variable for chironomid assemblage composition, consistent with earlier studies showing that oxygen availability, particularly deepwater oxygen concentration during summer months in stratified lakes, can have significant effects on the taxonomic composition of chironomid larvae (Little and Smol 2001; Verbruggen et al. 2011; Nevalainen and Luoto 2012; Lapellegerie et al. 2026). However, in our analyses, oxygen concentrations averaged across the entire water column explained more variance in chironomid assemblage composition than bottom‐water oxygen concentrations alone (Table 2). This likely reflects that many of the examined lakes are relatively shallow and experience frequent mixing and weak stratification, which limit hypolimnetic hypoxia. Under such conditions, bottom‐water oxygen concentrations measured under a brief period of thermal stratification may be less representative of oxygen conditions that influence chironomid assemblages than whole‐column conditions. Finally, water depth is also identified as a significant explanatory variable in our survey and has previously been recognized as a potentially important determinant of chironomid assemblage composition in lakes (Korhola et al. 2000; Engels and Cwynar 2011). However, as in other studies from the Alps (e.g., Lotter et al. 1997; Bigler et al. 2006), water depth appears to be a less critical variable affecting chironomid assemblages than temperature, DOC, nutrients, and associated variables.

### 4.2 Shifts in Chironomid Assemblages at Individual Study Lakes in the Past Decades

Although some records describing chironomid assemblage change over recent decades are available from mountain regions, many lack sufficient temporal resolution to reliably detect assemblage trends at decadal and subdecadal scales (Battarbee et al. 2002). Some studies report shifts in chironomid assemblages toward states indicative of higher temperatures, for example, in the Sierra Nevada, Spain (Jiménez‐Moreno et al. 2023, 2025), the Rocky Mountains, USA (Porinchu et al. 2017), or in the Austrian Alps (Ilyashuk et al. 2011). However, in other records, assemblage changes coinciding with recent decadal‐scale warming are less pronounced (e.g., Szabó et al. 2024) or difficult to detect (Von Gunten et al. 2008; Tombor et al. 2025). Changes in chironomid abundances between 1993/2002 and 2022–2023 (Figure 3), along with variations in DCCA axis 1 scores from multi‐variable and single‐variable analyses (Figure 5), reveal that distinct changes of individual chironomid taxa are apparent for some lakes, such as a pronounced decrease in *Micropsectra radialis*‐type at LUN and a major increase of *Tanytarsus lugens*‐type in PIS (Figure 4). For other sites and taxa, changes remain small, typically a few percent. However, despite the generally small amplitude of change, the majority of sites show consistent directional change.

In colder, oligotrophic lakes on the right side of the multivariable DCCA (e.g., PIS, MUR, LUN, SGR, SCE; Figure 4), assemblages are dominated by *Paracladius*, *T. lugens* ‐type, *M. radialis* ‐type, and *Pseudodiamesa*, previously described as dominating in high elevation Swiss lakes (Bigler et al. 2006; Heiri et al. 2011). In these lakes, a general trend shows decreasing abundances of *M. radialis* ‐type (e.g., 35% in PIS, 22% in MUR, 40% in LUN) with complete disappearance in some cases (e.g., SGR), whereas *T. lugens* ‐type shows increasing abundances (e.g., 49% in PIS, 22% in SGR) and new appearances (e.g., up to 35% in MUR). While *M. radialis* ‐type and *T. lugens* ‐type are both common in high alpine environments (e.g., Bigler et al. 2006; Ilyashuk et al. 2015), previous studies showed that *M. radialis* ‐type is often more abundant during colder periods than *T. lugens* ‐type (e.g., Ilyashuk et al. 2009, 2015). *T. lugens* ‐type is considered an oligo‐ to mesotrophic taxon (Saether 1979) and a cold stenotherm (Hofmann 1988), often found dominating in small shallow alpine lakes between 1800 and 2400 m a.s.l. in the Swiss Alps (Heiri and Lotter 2008). Comparatively, *M. radialis* ‐type is typically found in alpine lakes above 2000 m a.s.l. (Lotter et al. 1997; Heiri et al. 2011; Ilyashuk et al. 2011). Single‐variable DCCAs indicate that sites with decreasing abundances of *M. radialis* ‐type shift toward assemblage states typical of elevated DOC (PIS, MUR, SGR, SCE) and water temperature (PIS, MUR, and SGR; Figure 5B,C).

*Paracladius* shows variable trends across the coldest lakes, showing both increases (e.g., by 47% in LUN, 41% in SCE) and decreases (e.g., by 30% in SGR, 10% in MUR) in the modern samples. *Paracladius* tends to increase where *T. lugens* ‐type is absent or rare, whereas it often declines where *T. lugens* ‐type is becoming more abundant (e.g., SGR, SUV, MUR). This pattern suggests that the two taxa may interact through temperature‐driven dynamics or competition and is consistent with the cold‐stenothermous character of *Paracladius* (Walker et al. 1997) and the broader thermal tolerance of *T. lugens* ‐type (Heiri et al. 2011). In LUN, *Paracladius* increases in the modern sample, whereas *M. radialis* ‐type and *Pseudodiamesa* decrease strongly (Figure 3), ultimately shifting assemblage composition toward an overall state typical of warmer lakes (Figure 5). Similarly, *Pseudodiamesa*, a genus described as more cold‐adapted than *M. radialis* ‐type and tolerant of harsh climatic conditions (Ilyashuk et al. 2011), has declined in several lakes (e.g., PIS, LAN, IFF, SAG, and SCE), consistent with a broader shift towards more warm‐adapted assemblages under rising summer temperatures.

In the warmer, more DOC‐ and nutrient‐rich lakes on the left side of the DCCA (e.g., LUT, LIO, ENG, PIT, NAI, GUG; Figure 4), assemblages are more diverse than in colder, more oligotrophic lakes (Figure 3). Dominant taxa include *Chironomus anthracinus* ‐type, *Heterotrisscladius*, *Psectrocladius sordidellus* ‐type, and *Dicrotendipes notatus*‐type, typical of oligo‐ to mesotrophic lakes (Lotter et al. 1998), with broad thermal ranges across Swiss and Norwegian lakes (Heiri et al. 2011). Additionally, taxa tolerant of reduced oxygen conditions, such as *Chironomus*, *Procladius*, and *Glyptotendipes* (Brodersen and Quinlan 2006), tend to be more abundant at high‐DOC sites (CHA, LUT, RET, PIT). Additional taxa such as *Cladotanytarsus*, *Tanytarsus mendax* ‐type, and *Micropsectra insignilobus* ‐type occur mainly in temperate to mid‐elevation lakes (Saether 1979; Luoto 2009; Heiri et al. 2011). GUG shows the strongest shift towards assemblages typical of lower‐elevation and higher‐DOC conditions (Figure 5), with increased richness, declining *P. sordidellus* ‐type, and the appearance of *Parakiefferiella bathophila*‐type. As one of the shallowest lakes in the dataset (3.6 m), GUG is particularly sensitive to climatic fluctuations affecting ice‐cover duration, water chemistry, and habitat structure (Rouse et al. 1997; Pilla et al. 2020). Other trends in these lakes include increasing abundances of *T. mendax* ‐type (e.g., NAI, PIT), decreasing values of *T. lugens* ‐type (e.g., PIT, ENG, LUT) and *C. anthracinus* ‐type (PIT, LUT), as well as both increasing (ENG) and decreasing (NAI) abundances of *M. insignilobus* ‐type (Figure 3). Overall, these shifts reflect a general trend of warmer‐adapted taxa becoming increasingly common, while colder‐adapted taxa decline.

A subset of lakes (BAC, WAN, RET) exhibits shifts opposite to the overall warming trend, with more positive axis 1 values in the multivariable DCCA (Figure 5A). RET exhibits the strongest change (Figure 4), driven by taxa associated with lower DOC and increasing oxygen concentrations at higher elevations in our modern assemblage data (Figure 4). This shift corresponds to a marked decrease in *Einfeldia*, a littoral taxon typical of eutrophic lakes in the Swiss Alps (Pinder and Reiss 1983), and its replacement by more warm‐adapted littoral taxa that do not strongly deviate ecologically from *Einfeldia* (e.g., *Cladopelma lateralis*‐type, *Polypedilum nubeculosum* ‐type, *Endochironomus tendens*‐type, *Cladotanytarsus mancus* ‐type 1; Walker and Mathewes 1989; Walker and MacDonald 1995; Zheng et al. 2020). BAC and WAN show increases in *M. radialis* ‐type and declines in *P. sordidellus* ‐type, *M. insignilobus* ‐type, *H. marcidus* ‐type, and *Eukiefferiella*, consistent with shifts towards colder, high‐oxygen assemblages. BAC is managed for hydropower generation and has been reported to be affected by a decline in the water table during the winter months (Guthruf et al. 1999), which may explain unexpected assemblage changes. Overall, the trajectories observed in these lakes demonstrate that, while most lakes (15 out of 24) trend toward assemblages typical of warmer conditions, a subset of lakes may have developed assemblages typical of colder climatic conditions due to site‐specific or ecological factors.

### 4.3 Overall Trajectories of Change From 1993/2002 to 2022–2023

An evaluation of assemblage trajectories across our entire dataset between 1993/2002 and 2022–2023 indicates a clear shift toward chironomid assemblages typical of lower bottom‐water oxygen concentrations, higher DOC and nutrient concentrations, and higher temperatures over the past 2–3 decades in most lakes (Figure 4). Although DCCA trajectories vary among lakes, a Wilcoxon signed‐rank test (*p* = 0.011) indicates a significant overall trend toward assemblages typical of warmer conditions, and single‐variable DCCAs confirm shifts toward assemblages associated with higher temperature and DOC, demonstrating that the observed trends are not simply an artefact of the internal correlation structure of the dataset, but also apparent if variables are tested individually. We therefore conclude that our results provide robust evidence for a clear change in chironomid assemblage composition in the majority of alpine lakes in our study region as a response to recent warming. Indirectly, this strongly suggests that not only the aquatic insect assemblages at these sites but also Swiss mountain lake ecosystems as a whole are widely responding to increasing temperatures and variations in associated variables, such as DOC, nutrients, and oxygen concentrations. The observation that changes in chironomid assemblages vary distinctly between some sites suggests that individual lake responses to the observed warming trend in recent decades were strongly modulated by local factors. This high variability could be due to a combination of factors, including lake‐specific characteristics such as water depth, morphometry, and hydrology, which can influence temperature dynamics, oxygen levels, habitat stability, ice cover duration and ice‐off timing, as well as catchment‐related factors such as land use, permafrost, nutrient inputs, and DOC availability. Biotic interactions, including the presence of fish, may further shape local assemblage trajectories, leading to differing responses among lakes despite a common regional warming trend. While the exact reasons for stable or unexpected assemblage trajectories at individual sites remain unclear, local disturbances may explain some cases, such as fish introductions at TSC (Von Gunten et al. 2008) and hydropower‐related water‐level fluctuations at BAC (Guthruf et al. 1999). DOC concentrations were identified as one of the strongest explanatory variables for between‐lake variability in chironomid assemblages in our surface‐sediment dataset collected 2022–2023, consistent with earlier surveys of chironomid distributions in small alpine lakes (e.g., Heiri and Lotter 2008). This suggests that changes in DOC concentrations, or variables controlling DOC in lakes, such as catchment productivity, soil attributes, growing‐season length, in‐lake productivity, and lake nutrient concentrations, are among the primary ways in which warming climate conditions affect small Alpine lakes and their biotic communities.

Multi‐lake assessments comparable to our 24‐lake survey are rare. Kuefner, Hofmann, et al. (2020); Kuefner, Ossyssek, et al. (2020); and Kuefner et al. (2021) analysed diatom assemblage changes over recent decades across subsets of lakes (21–41 sites) in the Bavarian/Tyrolean Alps and documented shifts toward smaller, less‐silicified species, driven primarily by climate warming and modulated by lake‐specific conditions. Moser et al. (2019) reviewed palaeolimnological records and identified numerous studies showing that recent warming produced noticeable ecological changes in mountain lakes. In Andean and Tibetan lakes, increased water‐column stability under warming has altered algal communities and reduced primary production (Michelutti, Wolfe, et al. 2015; Michelutti, Cooke, et al. 2015; Labaj et al. 2017). Other studies show that glacier retreat modifies nutrient and DOC inputs to downstream lakes (Baron et al. 2009; Saros et al. 2010; Peter and Sommaruga 2016), while changing atmospheric deposition has driven long‐term shifts such as acidification‐recovery and nitrogen‐induced changes in phytoplankton (Wolfe et al. 2001; Kopáček et al. 2015). In an additional study, lake sediments from three Swiss mountain lakes further demonstrate how nutrients, fish predation, and climate change have jointly influenced lake ecosystems (Perga et al. 2015). Based on multiple proxies, it has been shown that climate warming has become an increasingly important driver of change in recent decades, although responses differed among lakes and taxa. For European mountain lakes, initiatives such as ALPE (Wathne et al. 1995; Battarbee et al. 2009), MOLAR (Battarbee et al. 2002, 2009), and EMERGE (Marchetto et al. 2008) examined decadal‐scale ecological change in the 1990s–2000s. Although these efforts provided extensive insights into pressures such as fish introductions and acid rain, the effects of climate change were difficult to quantify (Battarbee et al. 2002), partly because the temperature signature was weaker and less distinct in decades preceding these projects (Battarbee et al. 2002).

Our survey differs from earlier studies by focusing on a benthic deepwater indicator rarely examined in earlier large‐scale, multi‐lake studies of mountain regions, which often emphasized planktonic or littoral organisms such as diatoms or cladocerans (e.g., Michelutti, Wolfe, et al. 2015; Michelutti, Cooke, et al. 2015; Kuefner et al. 2021). Because benthic profundal communities are generally less affected by seasonal variability, wind‐driven mixing, and localized habitat conditions than many pelagic and littoral proxies, they may provide a clearer signal of long‐term climate‐related change (Bigler et al. 2006; Hausmann et al. 2011; Perga et al. 2015). In addition, because we compared two sets of surface sediment surveys from clearly defined sampling periods (1993/2002 and 2022–2023), our study is not affected by dating uncertainties and avoids the logistical challenges of developing numerous independent lake‐sediment geochronologies. Our study documents a statistically significant shift toward chironomid assemblages typical of warmer conditions over a wide range of lakes in the Swiss Alps, and therefore also provides evidence that these lakes and their biotic assemblages are reacting to ongoing climate warming. However, we also show that some sites exhibit little or even opposite assemblage changes over the past 2–3 decades, highlighting that studies focusing on a limited number of lakes may yield biased assessments of mountain lake responses to recent environmental change (Heino et al. 2021).

The overall trend across the 24 lakes indicated a shift toward chironomid assemblages typical of warmer climates, but the magnitude of change varied considerably among lakes. Although our study examines a relatively large number of sites, it lacks the necessary resolution and statistical power to determine whether the response to recent environmental change differs among lake types (e.g., with respect to water depth, nutrient concentrations, or catchment characteristics). Visual examination of the shift in axis 1 scores of our multi‐variable DCCA relative to elevation (Figures 5 and 6) suggests that changes towards assemblage states typical of higher temperatures and associated water chemistry conditions may be more pronounced at higher elevation, whereas assemblage shifts seem to be more variable at lower sites, consistent with pronounced summer warming documented at higher Alpine elevations (MRI EDW Working Group 2015; Kotlarski et al. 2023). Correlations between shifts in DCCA axis 1 scores and elevation or related catchment characteristics such as maximum catchment elevation or potential permafrost distribution, were only moderately strong and not statistically significant when all sites were examined. However, considerably stronger relationships emerged when only sites with relatively large shifts in DCCA axis 1 scores were examined (Figure 6). Particularly pronounced, largely unidirectional effects of recent warming on the highest‐elevation sites would agree with earlier studies suggesting that such lakes are strongly susceptible to changes in the alpine cryosphere, such as an increasing ice‐free period and melting of catchment permafrost (Noetzli et al. 2024), which may be linked to increased mobilization of DOC as well as alterations of the hydrochemical regimes (Vonk et al. 2015; Heffernan et al. 2024). In contrast, our lower‐elevation sites are located at elevations where changes in local human activities, including transhumance, pasturing, and tourism, may potentially obscure and modify the effects of warming temperatures, providing a potential explanation for a less pronounced and more diverse response of ecosystems to recent warming. Considering the limited size of our dataset and the presence of moderately strong but only marginally or non‐significant relationships of the amplitude of chironomid assemblage change with elevation and related variables, we believe an important next step would be to expand our study to a larger number of lakes. This would allow a more detailed assessment of the extent to which assemblage changes correlate with environmental conditions at the study lakes and reveal whether stronger, statistically significant relationships emerge if more sites are examined.

## 5 Conclusions

Our sampling campaign of 24 Swiss mountain lakes and the comparison of the results with earlier surveys provides a unique, multi‐decadal perspective on chironomid community dynamics in high‐elevation environments in the Alps. Directional shifts consistent with recent regional climatic warming were observed in most lakes. The replacement and reduction of cold‐stenothermic taxa by more generalist or warm‐associated taxa aligns with the well‐established sensitivity of chironomids to temperature. However, some lake systems exhibited little to no change, and others showed assemblage patterns suggesting that cold‐associated taxa remained resilient or even increased in abundance. These heterogeneous responses indicate that local conditions, such as catchment setting, hydrology, and organic matter dynamics, play a major role in modulating and potentially buffering climatic signals, leading to non‐uniform patterns of biological change across alpine landscapes. Overall, our study highlights that both large‐scale climatic drivers and local environmental heterogeneity shape chironomid assemblages in alpine environments. The variability of observed trajectories underscores the complexity of ecological responses to global change, and the necessity of multi‐lake, long‐term comparisons and monitoring to capture both regional trends and site‐specific variability. Most of our study sites show the expected directional change toward assemblages typical of higher temperatures, but several lakes show only a muted response. This can potentially be explained by extirpation and immigration of species lagging the climate warming at individual sites (Jackson and Sax 2010) and by local threshold effects not yet reached in the course of recent warming. This implies that our study, even though it documents clear evidence of early responses in aquatic insect assemblages in Swiss mountain lakes to increasing temperatures, may underestimate the eventual ecological consequences of climate warming on chironomid populations in these lakes. We therefore conclude that shifts in chironomid populations may increase in amplitude under continued warming in the Alps, as rising temperatures further affect mountain ecosystems and progressively cross critical thermal thresholds and tipping points. Sites that currently show only a weak or delayed response may undergo a more rapid and pronounced ecological reorganization in the coming decades, with consequences for biodiversity and ecosystem functioning.

## Author Contributions

**Maja Damber:** investigation, conceptualization, writing – original draft, formal analysis, writing – review and editing. **Laura Jiménez‐Liébanas:** writing – review and editing. **Carmen Docci:** formal analysis, writing – review and editing. **Pierre Lapellegerie:** conceptualization, investigation, formal analysis, writing – review and editing. **Juliane Krenz:** resources, supervision, writing – review and editing. **Nikolaus J. Kuhn:** resources, supervision. **Oliver Heiri:** funding acquisition, conceptualization, writing – original draft, writing – review and editing. **André F. Lotter:** resources, writing – review and editing. **Sandra O. Camara‐Brugger:** conceptualization, writing – review and editing. **Ghéreint Devillet:** conceptualization, investigation, writing – review and editing.

## Funding

This work was supported by Schweizerischer Nationalfonds zur Förderung der Wissenschaftlichen Forschung (200021_204222).

## Conflicts of Interest

The authors declare no conflicts of interest.

## Supporting information


**Table S1:** Summary of water chemistry analysis conducted by Amt für Umwelt und Energie of the Canton of Basel‐Stadt.

## Data Availability

The data that support the findings of this study are openly available in Zenodo at https://doi.org/10.5281/zenodo.20489929.

## References

[gcb70957-bib-0001] Adrian, R. , C. M. O'Reilly , H. Zagarese , et al. 2009. “Lakes as Sentinels of Climate Change.” Limnology and Oceanography 54: 2283–2297. 20396409 10.4319/lo.2009.54.6_part_2.2283 PMC2854826

[gcb70957-bib-0003] Baron, J. S. , T. M. Schmidt , and M. D. Hartman . 2009. “Climate‐Induced Changes in High Elevation Stream Nitrate Dynamics.” Global Change Biology 15, no. 7: 1777–1789.

[gcb70957-bib-0004] Battarbee, R. W. , M. Kernan , and N. Rose . 2009. “Threatened and Stressed Mountain Lakes of Europe: Assessment and Progress.” Aquatic Ecosystem Health and Management 12, no. 2: 118–128.

[gcb70957-bib-0005] Battarbee, R. W. , R. Thompson , J. Catalan , J.‐A. Grytnes , and H. J. B. Birks . 2002. “Climate Variability and Ecosystem Dynamics of Remote Alpine and Arctic Lakes: The MOLAR Project.” Journal of Paleolimnology 28: 1–6.

[gcb70957-bib-0006] Bigler, C. , O. Heiri , R. Krskova , A. F. Lotter , and M. Sturm . 2006. “Distribution of Diatoms, Chironomids and Cladocera in Surface Sediments of Thirty Mountain Lakes in South‐Eastern Switzerland.” Aquatic Sciences 68, no. 2: 154–171.

[gcb70957-bib-0007] Bolland, A. , O. A. Kern , A. Koutsodendris , J. Pross , and O. Heiri . 2022. “Chironomid‐Inferred Summer Temperature Development During the Late Rissian Glacial, Eemian Interglacial and Earliest Würmian Glacial at Füramoos, Southern Germany.” Boreas 51, no. 2: 496–516.

[gcb70957-bib-0134] Bolland, A. , F. Rey , E. Gobet , W. Tinner , and O. Heiri . 2020. “Summer Temperature Development 18,000–14,000 cal. BP Recorded by a new Chironomid Record from Burgäschisee, Swiss Plateau.” Quaternary Science Reviews 243: 106484.

[gcb70957-bib-0008] Brodersen, K. P. , and C. Lindegaard . 1999. “Classification, Assessment and Trophic Reconstruction of Danish Lakes Using Chironomids.” Freshwater Biology 42, no. 1: 143–157.

[gcb70957-bib-0009] Brodersen, K. P. , and R. Quinlan . 2006. “Midges as Palaeoindicators of Lake Productivity, Eutrophication and Hypolimnetic Oxygen.” Quaternary Science Reviews 25, no. 15–16: 1995–2012.

[gcb70957-bib-0011] Brooks, S. J. , P. G. Langdon , and O. Heiri . 2007. The Identification and Use of Palaearctic Chironomidae Larvae in Palaeoecology. Quaternary Research Association.

[gcb70957-bib-0012] Catalan, J. , M. G. Barbieri , F. Bartumeus , et al. 2009. “Ecological Thresholds in European Alpine Lakes.” Freshwater Biology 54: 2494–2517.

[gcb70957-bib-0013] Demars, B. O. , J. L. Kemp , B. Marteau , N. Friberg , and B. Thornton . 2021. “Stream Macroinvertebrates and Carbon Cycling in Tangled Food Webs.” Ecosystems 24, no. 8: 1944–1961.

[gcb70957-bib-0014] Eggermont, H. , and O. Heiri . 2012. “The Chironomid‐Temperature Relationship: Expression in Nature and Palaeoenvironmental Implications.” Biological Reviews 87, no. 2: 430–456. 22032243 10.1111/j.1469-185X.2011.00206.x

[gcb70957-bib-0015] Engels, S. , and L. C. Cwynar . 2011. “Changes in Fossil Chironomid Remains Along a Depth Gradient: Evidence for Common Faunal Thresholds Within Lakes.” Hydrobiologia 665, no. 1: 15–38.

[gcb70957-bib-0017] Federal Office of Topography swisstopo . 2023. “SwissALTI^Regio^: Small‐Scale Digital Elevation Model of Switzerland and Surrounding Regions [Dataset].” https://www.swisstopo.admin.ch/en/height‐model‐swissaltiregio.

[gcb70957-bib-0018] Federal Office of Topography swisstopo . 2024. “SwissALTI3D: High‐Precision Digital Elevation Model of Switzerland [Dataset].” https://www.swisstopo.admin.ch/en/height‐model‐swissalti3d.

[gcb70957-bib-0019] Federal Office of Topography swisstopo . 2026a. “Geological Atlas of Switzerland 1:25,000 (GA25).” https://map.geo.admin.ch/#/map?layers=ch.swisstopo.geologie‐geologischer_atlas.

[gcb70957-bib-0020] Federal Office of Topography swisstopo . 2026b. “Potential Permafrost Distribution Map [Web Map Service].” https://map.geo.admin.ch/#/map?layers=ch.bafu.permafrost.

[gcb70957-bib-0021] Fischer, A. M. , K. M. Strassmann , M. Croci‐Maspoli , et al. 2022. “Climate Scenarios for Switzerland CH2018–Approach and Implications.” Climate Services 26: 100288.

[gcb70957-bib-0022] Forsström, L. , T. Roiha , and M. Rautio . 2013. “Responses of Microbial Food Web to Increased Allochthonous DOM in an Oligotrophic Subarctic Lake.” Aquatic Microbial Ecology 68, no. 2: 171–184.

[gcb70957-bib-0023] Foucault, Q. , A. Wieser , A. M. Waldvogel , B. Feldmeyer , and M. Pfenninger . 2018. “Rapid Adaptation to High Temperatures in *Chironomus riparius* .” Ecology and Evolution 8, no. 24: 12780–12789. 30619582 10.1002/ece3.4706 PMC6308882

[gcb70957-bib-0024] Frossard, V. , P. Sabatier , R. Bruel , et al. 2023. “Intense Touristic Activities Exceed Climate Change to Shape Aquatic Communities in a Mountain Lake.” Aquatic Sciences 85, no. 3: 71. 37192889 10.1007/s00027-023-00968-6 PMC10157129

[gcb70957-bib-0025] Gannon, J. E. 1971. “Two Counting Cells for the Enumeration of Zooplankton Micro‐Crustacea.” Transactions of the American Microscopical Society 90: 486–490.

[gcb70957-bib-0026] Gobiet, A. , S. Kotlarski , M. Beniston , G. Heinrich , J. Rajczak , and M. Stoffel . 2014. “21st Century Climate Change in the European Alps—A Review.” Science of the Total Environment 493: 1138–1151. 23953405 10.1016/j.scitotenv.2013.07.050

[gcb70957-bib-0027] Guthruf, J. , K. Guthruf‐Seiler , and M. Zeh . 1999. Kleinseen im Kanton Bern. Amt für Gewässerschutz und Abfallwirtschaft des Kantons Bern, Gewässer‐ und Bodenschutzlabor.

[gcb70957-bib-0028] Hammer, Ø. , and D. A. Harper . 2001. “Past: Paleontological Statistics Software Package for Educaton and Data Analysis.” Palaeontologia Electronica 4, no. 1: 9.

[gcb70957-bib-0029] Hausmann, S. , I. Larocque‐Tobler , P. J. Richard , R. Pienitz , G. St‐Onge , and F. Fye . 2011. “Diatom‐Inferred Wind Activity at Lac du Sommet, Southern Québec, Canada: A Multiproxy Paleoclimate Reconstruction Based on Diatoms, Chironomids and Pollen for the Past 9500 Years.” Holocene 21, no. 6: 925–938.

[gcb70957-bib-0030] Heffernan, L. , D. N. Kothawala , and L. J. Tranvik . 2024. “Terrestrial Dissolved Organic Carbon in Northern Permafrost.” Cryosphere 18, no. 3: 1443–1465.

[gcb70957-bib-0031] Heino, J. , J. Alahuhta , L. M. Bini , et al. 2021. “Lakes in the Era of Global Change: Moving Beyond Single‐Lake Thinking in Maintaining Biodiversity and Ecosystem Services.” Biological Reviews 96, no. 1: 89–106. 32869448 10.1111/brv.12647

[gcb70957-bib-0032] Heiri, O. , S. J. Brooks , H. J. B. Birks , and A. F. Lotter . 2011. “A 274‐Lake Calibration Data‐Set and Inference Model for Chironomid‐Based Summer Air Temperature Reconstruction in Europe.” Quaternary Science Reviews 30, no. 23–24: 3445–3456.

[gcb70957-bib-0033] Heiri, O. , and S. E. Engels . 2026. “Combine, Assign or Delete? How to Resolve Different Levels of Taxonomic Identification in Chironomid Datasets.” Journal of Paleolimnology 74: 6. 41924454 10.1007/s10933-026-00387-1 PMC13035590

[gcb70957-bib-0034] Heiri, O. , and A. F. Lotter . 2001. “Effect of Low Count Sums on Quantitative Environmental Reconstructions: An Example Using Subfossil Chironomids.” Journal of Paleolimnology 26, no. 3: 343–350.

[gcb70957-bib-0035] Heiri, O. , and A. F. Lotter . 2003. “9000 Years of Chironomid Assemblage Dynamics in an Alpine Lake: Long‐Term Trends, Sensitivity to Disturbance, and Resilience of the Fauna.” Journal of Paleolimnology 30: 273–289.

[gcb70957-bib-0036] Heiri, O. , and A. F. Lotter . 2008. “Chironomidae (Diptera) in Alpine Lakes: A Study of Subfossil Assemblages in Lake Surface Sediments.” Boletim Do Museu Municipal Do Funchal (História Natural) 13: 177–184.

[gcb70957-bib-0037] Heiri, O. , and A. F. Lotter . 2010. “How Does Taxonomic Resolution Affect Chironomid‐Based Temperature Reconstruction?” Journal of Paleolimnology 44, no. 2: 589–601.

[gcb70957-bib-0038] Hill, M. O. , and H. G. Gauch Jr. 1980. “Detrended Correspondence Analysis: An Improved Ordination Technique.” Vegetatio 42, no. 1: 47–58.

[gcb70957-bib-0133] Hofmann, W. 1986. “Chironomid Analysis.” In Handbook of Holocene Palaeoecology and Paleohydrology, edited by B. E. Berglund , 715–727. John Wiley & Sons.

[gcb70957-bib-0039] Hofmann, W. 1988. “The Significance of Chironomid Analysis (Insecta: Diptera) for Paleolimnological Research.” Palaeogeography, Palaeoclimatology, Palaeoecology 62, no. 1–4: 501–509.

[gcb70957-bib-0040] Ilyashuk, B. , E. Gobet , O. Heiri , et al. 2009. “Lateglacial Environmental and Climatic Changes at the Maloja Pass, Central Swiss Alps, as Recorded by Chironomids and Pollen.” Quaternary Science Reviews 28, no. 13–14: 1340–1353.

[gcb70957-bib-0041] Ilyashuk, E. A. , B. P. Ilyashuk , W. Tylmann , K. A. Koinig , and R. Psenner . 2015. “Biodiversity Dynamics of Chironomid Midges in High‐Altitude Lakes of the Alps Over the Past Two Millennia.” Insect Conservation and Diversity 8, no. 6: 547–561.

[gcb70957-bib-0042] Ilyashuk, E. A. , K. A. Koinig , O. Heiri , B. P. Ilyashuk , and R. Psenner . 2011. “Holocene Temperature Variations at a High‐Altitude Site in the Eastern Alps: A Chironomid Record From Schwarzsee Ob Sölden, Austria.” Quaternary Science Reviews 30, no. 1–2: 176–191. 21317974 10.1016/j.quascirev.2010.10.008 PMC3021123

[gcb70957-bib-0043] Jackson, S. T. , and D. F. Sax . 2010. “Balancing Biodiversity in a Changing Environment: Extinction Debt, Immigration Credit and Species Turnover.” Trends in Ecology & Evolution 25, no. 3: 153–160. 19879014 10.1016/j.tree.2009.10.001

[gcb70957-bib-0044] Jiménez‐Moreno, G. , O. Heiri , A. García‐Alix , et al. 2023. “Holocene Summer Temperature Reconstruction Based on a Chironomid Record From Sierra Nevada, Southern Spain.” Quaternary Science Reviews 319: 108343.

[gcb70957-bib-0045] Jiménez‐Moreno, G. , N. Prat , O. Heiri , et al. 2025. “Chironomid‐Based Holocene Summer Temperature Dynamics From Southern Spain.” Quaternary Science Reviews 369: 109647.

[gcb70957-bib-0046] Kopáček, J. , S. Bičárová , J. Hejzlar , et al. 2015. “Catchment Biogeochemistry Modifies Long‐Term Effects of Acidic Deposition on Chemistry of Mountain Lakes.” Biogeochemistry 125, no. 3: 315–335.

[gcb70957-bib-0047] Korhola, A. , H. Olander , and T. Blom . 2000. “Cladoceran and Chironomid Assemblages as Qualitative Indicators of Water Depth in Subarctic Fennoscandian Lakes.” Journal of Paleolimnology 24, no. 1: 43–54.

[gcb70957-bib-0048] Kotlarski, S. , A. Gobiet , S. Morin , M. Olefs , J. Rajczak , and R. Samacoïts . 2023. “21st Century Alpine Climate Change.” Climate Dynamics 60, no. 1: 65–86.

[gcb70957-bib-0049] Kowalyk, H. E. 1985. “The Larval Cephalic Setae in the Tanypodinae (Diptera: Chironomidae) and Their Importance in Generic Determinations.” Canadian Entomologist 117, no. 1: 67–106.

[gcb70957-bib-0050] Kuefner, W. , A. Hofmann , S. Ossyssek , N. Dubois , J. Geist , and U. Raeder . 2020. “Composition of Highly Diverse Diatom Community Shifts as Response to Climate Change: A Down‐Core Study of 23 Central European Mountain Lakes.” Ecological Indicators 117: 106590.

[gcb70957-bib-0051] Kuefner, W. , A. M. Hofmann , J. Geist , N. Dubois , and U. Raeder . 2021. “Algal Community Change in Mountain Lakes of the Alps Reveals Effects of Climate Warming and Shifting Treelines.” Journal of Phycology 57, no. 4: 1266–1283. 33751611 10.1111/jpy.13163

[gcb70957-bib-0052] Kuefner, W. , S. Ossyssek , J. Geist , and U. Raeder . 2020. “The Silicification Value: A Novel Diatom‐Based Indicator to Assess Climate Change in Freshwater Habitats.” Diatom Research 35, no. 1: 1–16.

[gcb70957-bib-0053] Labaj, A. L. , N. Michelutti , and J. P. Smol . 2017. “Changes in Cladoceran Assemblages From Tropical High Mountain Lakes During Periods of Recent Climate Change.” Journal of Plankton Research 39, no. 2: 211–219.

[gcb70957-bib-0054] Lapellegerie, P. , S. Breu , L. Wick , et al. 2026. “Dynamic Deepwater Invertebrate Populations Challenge the Concept of Oxygen‐Rich Reference Conditions for European Lakes.” Communications Earth & Environment 7: 301.

[gcb70957-bib-0055] Larocque, I. 2001. “How Many Chironomid Head Capsules Are Enough? A Statistical Approach to Determine Sample Size for Palaeoclimatic Reconstructions.” Palaeogeography, Palaeoclimatology, Palaeoecology 172, no. 1–2: 133–142.

[gcb70957-bib-0056] Larocque, I. , R. I. Hall , and E. Grahn . 2001. “Chironomids as Indicators of Climate Change: A 100‐Lake Training Set From a Subarctic Region of Northern Sweden (Lapland).” Journal of Paleolimnology 26, no. 3: 307–322.

[gcb70957-bib-0057] Larocque, I. , R. Pienitz , and N. Rolland . 2006. “Factors Influencing the Distribution of Chironomids in Lakes Distributed Along a Latitudinal Gradient in Northwestern Quebec, Canada.” Canadian Journal of Fisheries and Aquatic Sciences 63, no. 6: 1286–1297.

[gcb70957-bib-0060] Lepori, F. , B. Lucchini , C. Capelli , and F. Rotta . 2022. “Mesotrophy Is Not Enough: Re‐Assessing Phosphorus Objectives for the Restoration of a Deep Alpine Lake (Lake Lugano, Switzerland and Italy).” Advances in Oceanography and Limnology 13, no. 2: 11061.

[gcb70957-bib-0061] Little, J. L. , and J. P. Smol . 2001. “A Chironomid‐Based Model for Inferring Late‐Summer Hypolimnetic Oxygen in Southeastern Ontario Lakes.” Journal of Paleolimnology 26, no. 3: 259–270.

[gcb70957-bib-0062] Lotter, A. F. , H. J. B. Birks , W. Hofmann , and A. Marchetto . 1997. “Modern Diatom, Cladocera, Chironomid, and Chrysophyte Cyst Assemblages as Quantitative Indicators for the Reconstruction of Past Environmental Conditions in the Alps. I. Climate.” Journal of Paleolimnology 18, no. 4: 395–420.

[gcb70957-bib-0063] Lotter, A. F. , H. J. B. Birks , W. Hofmann , and A. Marchetto . 1998. “Modern Diatom, Cladocera, Chironomid, and Chrysophyte Cyst Assemblages as Quantitative Indicators for the Reconstruction of Past Environmental Conditions in the Alps. II. Nutrients.” Journal of Paleolimnology 19, no. 4: 443–463.

[gcb70957-bib-0064] Lotter, A. F. , O. Heiri , W. Hofmann , et al. 2006. “Holocene Timber‐Line Dynamics at Bachalpsee, a Lake at 2265 m Asl in the Northern Swiss Alps.” Vegetation History and Archaeobotany 15, no. 4: 295–307.

[gcb70957-bib-0065] Lotter, A. F. , W. Hofmann , C. Kamenik , et al. 2000. “Sedimentological and Biostratigraphical Analyses of Short Sediment Cores From Hagelseewli (2339 m Asl) in the Swiss Alps.” Journal of Limnology 59, no. 1: 53–64.

[gcb70957-bib-0066] Luoto, T. P. 2009. “Subfossil Chironomidae (Insecta: Diptera) Along a Latitudinal Gradient in Finland: Development of a New Temperature Inference Model.” Journal of Quaternary Science 24, no. 2: 150–158.

[gcb70957-bib-0067] Luoto, T. P. 2011. “The Relationship Between Water Quality and Chironomid Distribution in Finland—A New Assemblage‐Based Tool for Assessments of Long‐Term Nutrient Dynamics.” Ecological Indicators 11, no. 2: 255–262.

[gcb70957-bib-0068] Luoto, T. P. , M. V. Rantala , A. Galkin , M. Rautio , and L. Nevalainen . 2016. “Environmental Determinants of Chironomid Communities in Remote Northern Lakes Across the Treeline–Implications for Climate Change Assessments.” Ecological Indicators 61: 991–999.

[gcb70957-bib-0069] Marchetto, A. , M. Rogora , A. Boggero , et al. 2008. “Response of Alpine Lakes to Major Environmental Gradients, as Detected Through Planktonic, Benthic and Sedimentary Assemblages.” Advances in Limnology 62: 389–410.

[gcb70957-bib-0070] Marziali, L. , and B. Rossaro . 2013. “Response of Chironomid Species (Diptera, Chironomidae) to Water Temperature: Effects on Species Distribution in Specific Habitats.” Journal of Entomological and Acarological Research 45, no. 2: e14.

[gcb70957-bib-0072] Michelutti, N. , C. A. Cooke , W. O. Hobbs , and J. P. Smol . 2015. “Climate‐Driven Changes in Lakes From the Peruvian Andes.” Journal of Paleolimnology 54, no. 1: 153–160.

[gcb70957-bib-0073] Michelutti, N. , A. P. Wolfe , C. A. Cooke , W. O. Hobbs , M. Vuille , and J. P. Smol . 2015. “Climate Change Forces New Ecological States in Tropical Andean Lakes.” PLoS One 10, no. 2: 115338. 10.1371/journal.pone.0115338 PMC4315470 25647018

[gcb70957-bib-0074] Moser, K. A. , J. S. Baron , J. Brahney , et al. 2019. “Mountain Lakes: Eyes on Global Environmental Change.” Global and Planetary Change 178: 77–95.

[gcb70957-bib-0075] MRI EDW Working Group . 2015. “Elevation‐Dependent Warming in Mountain Regions of the World.” Nature Climate Change 5: 424–430.

[gcb70957-bib-0136] Müller, B. , A. F. Lotter , M. Sturm , and A. Ammann . 1998. “Influence of Catchment Quality and Altitude on the Water and Sediment Composition of 68 Small Lakes in Central Europe.” Aquatic Sciences 60, no. 4: 316–337.

[gcb70957-bib-0076] Nevalainen, L. , and T. P. Luoto . 2012. “Faunal (Chironomidae, Cladocera) Responses to Post‐Little Ice Age Climate Warming in the High Austrian Alps.” Journal of Paleolimnology 48, no. 4: 711–724.

[gcb70957-bib-0077] Nigrelli, G. , and M. Chiarle . 2023. “1991–2020 Climate Normal in the European Alps: Focus on High‐Elevation Environments.” Journal of Mountain Science 20, no. 8: 2149–2163.

[gcb70957-bib-0079] Noetzli, J. , K. Isaksen , J. Barnett , et al. 2024. “Enhanced Warming of European Mountain Permafrost in the Early 21st Century.” Nature Communications 15, no. 1: 10508. 10.1038/s41467-024-54831-9 PMC11631975 39658603

[gcb70957-bib-0080] Nyman, M. , A. Korhola , and S. J. Brooks . 2005. “The Distribution and Diversity of Chironomidae (Insecta: Diptera) in Western Finnish Lapland, With Special Emphasis on Shallow Lakes.” Global Ecology and Biogeography 14, no. 2: 137–153.

[gcb70957-bib-0082] Oksanen, J. 2016. “Design Decisions and Implementation Details in Vegan.” Vignette of the Package Vegan. R Package Version.

[gcb70957-bib-0083] Oleksy, I. A. , J. S. Baron , P. R. Leavitt , and S. A. Spaulding . 2020. “Nutrients and Warming Interact to Force Mountain Lakes Into Unprecedented Ecological States.” Proceedings of the Royal Society B 287: 20200304. 32635862 10.1098/rspb.2020.0304 PMC7423480

[gcb70957-bib-0084] Pastorino, P. , and M. Prearo . 2020. “High‐Mountain Lakes, Indicators of Global Change: Ecological Characterization and Environmental Pressures.” Diversity 12, no. 6: 260.

[gcb70957-bib-0085] Perga, M. E. , V. Frossard , J. P. Jenny , et al. 2015. “High‐Resolution Paleolimnology Opens New Management Perspectives for Lakes Adaptation to Climate Warming.” Frontiers in Ecology and Evolution 3: 72.

[gcb70957-bib-0087] Peter, H. , and R. Sommaruga . 2016. “Shifts in Diversity and Function of Lake Bacterial Communities Upon Glacier Retreat.” ISME Journal 10, no. 7: 1545–1554. 26771929 10.1038/ismej.2015.245 PMC4852812

[gcb70957-bib-0088] Pilla, R. M. , C. E. Williamson , B. V. Adamovich , et al. 2020. “Deeper Waters Are Changing Less Consistently Than Surface Waters in a Global Analysis of 102 Lakes.” Scientific Reports 10, no. 1: 20514. 33239702 10.1038/s41598-020-76873-x PMC7688658

[gcb70957-bib-0089] Pinder, L. C. R. , and F. Reiss . 1983. “The Larvae of Chironominae (Diptera: Chironomidae) of the Holarctic Region. Keys and Diagnoses.” Entomologica Scandinavica Supplementum 19: 293–435.

[gcb70957-bib-0090] Porinchu, D. F. , D. R. Haskett , and S. A. Reinemann . 2017. “Biostratigraphic Evidence of Human Modification of High Elevation Aquatic Ecosystems in the Intermountain West of the United States.” Anthropocene 20: 37–47.

[gcb70957-bib-0091] Quinlan, R. , and J. P. Smol . 2001. “Setting Minimum Head Capsule Abundance and Taxa Deletion Criteria in Chironomid‐Based Inference Models.” Journal of Paleolimnology 26, no. 3: 327–342.

[gcb70957-bib-0092] Renberg, I. , and H. Hansson . 2008. “The HTH Sediment Corer.” Journal of Paleolimnology 40, no. 2: 655–659.

[gcb70957-bib-0093] Rieradevall, M. , and S. J. Brooks . 2001. “An Identification Guide to Subfossil Tanypodinae Larvae (Insecta: Diptera: Chrironomidae) Based on Cephalic Setation.” Journal of Paleolimnology 25, no. 1: 81–99.

[gcb70957-bib-0094] Rogora, M. , L. Somaschini , A. Marchetto , R. Mosello , G. A. Tartari , and L. Paro . 2020. “Decadal Trends in Water Chemistry of Alpine Lakes in Calcareous Catchments Driven by Climate Change.” Science of the Total Environment 708: 135180. 31812417 10.1016/j.scitotenv.2019.135180

[gcb70957-bib-0135] Rottler, E. , C. Kormann , T. Francke , and A. Bronstert . 2019. “Elevation‐Dependent Warming in the Swiss Alps 1981–2017: Features, Forcings and Feedbacks.” International Journal of Climatology 39, no. 5: 2556–2568.

[gcb70957-bib-0095] Rouse, W. R. , M. S. Douglas , R. E. Hecky , et al. 1997. “Effects of Climate Change on the Freshwaters of Arctic and Subarctic North America.” Hydrological Processes 11, no. 8: 873–902.

[gcb70957-bib-0096] Saether, O. A. 1979. “Chironomid Communities as Water Quality Indicators.” Ecography 2, no. 2: 65–74.

[gcb70957-bib-0097] Saros, J. E. , K. C. Rose , D. W. Clow , et al. 2010. “Melting Alpine Glaciers Enrich High‐Elevation Lakes With Reactive Nitrogen.” Environmental Science and Technology 44, no. 13: 4891–4896. 20527763 10.1021/es100147j

[gcb70957-bib-0098] Schär, C. , T. D. Davies , C. Frei , et al. 1998. “Current Alpine climate.” In Views From the Alps: Regional Perspectives on Climate Change, edited by P. Cebon , U. Dahinden , S. Davies , et al. MIT Press.

[gcb70957-bib-0099] Schindler, D. W. 1977. “Evolution of Phosphorus Limitation in Lakes: Natural Mechanisms Compensate for Deficiencies of Nitrogen and Carbon in Eutrophied Lakes.” Science 195, no. 4275: 260–262. 17787798 10.1126/science.195.4275.260

[gcb70957-bib-0100] Schmid, P. E. 1993. “A Key to the Larval Chironomidae and Their Instars From Austrian Danube Region Streams and Rivers: With Particular Reference to a Numerical Taxonomic Approach.” Part I: Diamesinae, Prodiamesinae and Orthocladiinae. Federal Institute for Water Quality, Vienna.

[gcb70957-bib-0102] Schwörer, C. , P. Kaltenrieder , L. Glur , et al. 2014. “Holocene Climate, Fire and Vegetation Dynamics at the Treeline in the Northwestern Swiss Alps.” Vegetation History and Archaeobotany 23, no. 5: 479–496.

[gcb70957-bib-0104] Šmilauer, P. , and J. Lepš . 2014. Multivariate Analysis of Ecological Data Using CANOCO 5. 2nd ed. Cambridge University Press.

[gcb70957-bib-0106] Suranyi, T. , J. Talbot , D. Francis , et al. 2025. “Chironomid Assemblages in Surface Sediments From 182 Lakes Across New England and Eastern Canada: Development and Validation of a New Summer Temperature Transfer Function.” Quaternary Science Reviews 357: 109333.

[gcb70957-bib-0108] Szabó, Z. , K. Buczkó , J. L. Korponai , et al. 2024. “Two Chironomid‐Inferred Mean July Air Temperature Reconstructions in the South Carpathian Mountains Over the Last 2000 Years.” Holocene 34, no. 7: 987–1004.

[gcb70957-bib-0109] Tarrats Sada, P. , M. Caņedo‐Argüelles , M. Rieradevall i Sant , and N. Prat i Fornells . 2017. “Chironomid Communities as Indicators of Local and Global Changes in an Oligotrophic High Mountain Lake (Enol Lake, Northwestern Spain).” Journal of Limnology 76, no. 2: 355–365.

[gcb70957-bib-0110] ter Braak, C. J. 1990. “Update Notes: CANOCO Version 3.10.” Agricultural Mathematics Group, Wageningen: 1–35.

[gcb70957-bib-0111] ter Braak, C. J. , and I. C. Prentice . 1988. “A Theory of Gradient Analysis.” Advances in Ecological Research 18: 271–317.

[gcb70957-bib-0112] Tiberti, R. , F. Dory , F. Arthaud , et al. 2025. “Long‐Term Changes of Zooplankton in Alpine Lakes Result From a Combination of Local and Global Threats.” Biological Conservation 308: 111222.

[gcb70957-bib-0113] Tombor, E. , J. L. Korponai , R. Begy , et al. 2025. “Resilience of Alpine Lake Macroinvertebrate Communities to Climate Change: A View From the South Carpathian Mountains.” Hydrobiologia 852: 1–26.

[gcb70957-bib-0114] Verbruggen, F. , O. Heiri , J. J. Meriläinen , and A. F. Lotter . 2011. “Subfossil Chironomid Assemblages in Deep, Stratified European Lakes: Relationships With Temperature, Trophic State and Oxygen.” Freshwater Biology 56, no. 3: 407–423.

[gcb70957-bib-0116] Vitasse, Y. , S. Ursenbacher , G. Klein , et al. 2021. “Phenological and Elevational Shifts of Plants, Animals and Fungi Under Climate Change in the European Alps.” Biological Reviews 96, no. 5: 1816–1835. 33908168 10.1111/brv.12727

[gcb70957-bib-0117] Von Gunten, L. , O. Heiri , C. Bigler , et al. 2008. “Seasonal Temperatures for the Past ∼400 Years Reconstructed From Diatom and Chironomid Assemblages in a High‐Altitude Lake (Lej da la Tscheppa, Switzerland).” Journal of Paleolimnology 39, no. 3: 283–299.

[gcb70957-bib-0118] Vonk, J. E. , S. E. Tank , W. B. Bowden , et al. 2015. “Reviews and Syntheses: Effects of Permafrost Thaw on Arctic Aquatic Ecosystems.” Biogeosciences 12, no. 23: 7129–7167.

[gcb70957-bib-0121] Walker, I. R. , A. J. Levesque , L. C. Cwynar , and A. F. Lotter . 1997. “An Expanded Surface‐Water Palaeotemperature Inference Model for Use With Fossil Midges From Eastern Canada.” Journal of Paleolimnology 18, no. 2: 165–178.

[gcb70957-bib-0122] Walker, I. R. , and G. M. MacDonald . 1995. “Distributions of Chironomidae (Insecta: Diptera) and Other Freshwater Midges With Respect to Treeline, Northwest Territories, Canada.” Arctic and Alpine Research 27, no. 3: 258–263.

[gcb70957-bib-0123] Walker, I. R. , and R. W. Mathewes . 1989. “Chironomidae (Diptera) Remains in Surficial Lake Sediments From the Canadian Cordillera: Analysis of the Fauna Across an Altitudinal Gradient.” Journal of Paleolimnology 2, no. 1: 61–80.

[gcb70957-bib-0124] Walker, I. R. , J. P. Smol , D. R. Engstrom , and H. J. B. Birks . 1991. “An Assessment of Chironomidae as Quantitative Indicators of Past Climatic Change.” Canadian Journal of Fisheries and Aquatic Sciences 48, no. 6: 975–987.

[gcb70957-bib-0125] Wathne, B. M. , S. T. Patrick , D. Monteith , and H. Barth . 1995. “AL: PE‐Acidification of Mountain Lakes: Paleolimnology and Ecology.” AL: PE 1 Report for the Period April 1991–April 1993. European Commission. Ecosystem Research Report Vol. 9, Luxembourg.

[gcb70957-bib-0126] Weckström, K. , J. Weckström , K. Huber , et al. 2016. “Impacts of Climate Warming on Alpine Lake Biota Over the Past Decade.” Arctic, Antarctic, and Alpine Research 48, no. 2: 361–376.

[gcb70957-bib-0127] Wiederholm, T. 1983. “Chironomidae of the Holarctic Region. Keys and Diagnoses. Part 1: Larva.” Entomologica Scandinavica 19: 1–457.

[gcb70957-bib-0128] Wilson, S. E. , and K. Gajewski . 2004. “Modern Chironomid Assemblages and Their Relationship to Physical and Chemical Variables in Southwest Yukon and Northern British Columbia Lakes.” Arctic, Antarctic, and Alpine Research 36, no. 4: 446–455.

[gcb70957-bib-0129] Wolfe, A. P. , J. S. Baron , and R. J. Cornett . 2001. “Anthropogenic Nitrogen Deposition Induces Rapid Ecological Changes in Alpine Lakes of the Colorado Front Range (USA).” Journal of Paleolimnology 25, no. 1: 1–7.

[gcb70957-bib-0130] Woolway, R. I. , S. Sharma , G. A. Weyhenmeyer , et al. 2021. “Phenological Shifts in Lake Stratification Under Climate Change.” Nature Communications 12, no. 1: 2318. 10.1038/s41467-021-22657-4 PMC8055693 33875656

[gcb70957-bib-0131] Zheng, T. , Y. Cao , J. Peng , X. Bai , and X. Chen . 2020. “Effects of Climate Warming and Nitrogen Deposition on Subtropical Montane Ponds (Central China) Over the Last Two Centuries: Evidence From Subfossil Chironomids.” Environmental Pollution 262: 114256. 32126441 10.1016/j.envpol.2020.114256

