# Supplementary material for: Widespread Aquatic Insect Responses to Recent Warming in Swiss Mountain Lakes
Source: Glob Chang Biol. 2026 Jun 8;32(6):e70957. doi: 10.1111/gcb.70957 (PMC13244191; doi:10.1111/gcb.70957)
Supplement: Supplementary file 1 — Table S1: Summary of water chemistry analysis conducted by Amt für Umwelt und Energie of the Canton of Basel‐Stadt. [file GCB-32-e70957-s001.docx]

**SUPPLEMENTARY MATERIAL**

Table S1. Summary of water chemistry analysis conducted by Amt für Umwelt und Energie of the Canton of Basel-Stadt.

| **Analysis** | **Reference** | **Detection limt** | **Unit** |
| --- | --- | --- | --- |
| Photometry | DIN 38 404-3 |  |  |
| *SAK-254* |  | *0.03* | *1 m^-1^* |
| *SAK-436* |  | *0.03* | *1 m^-1^* |
|  |  |  |  |
| Combustion IR | DIN EN 1484; DIN EN  12260_2003 |  |  |
|  |  |  |  |
| *DOC* |  | *0.2* | *mg L^-1^* |
| *Total nitrogen* |  | *0.05* | *mg L^-1^* |
| *TOC* |  | *0.25* | *mg L^-1^* |
|  |  |  |  |
| Photometry | DIN EN ISO 6878:2004 |  |  |
| *Total phosphorus* |  | *0.003* | *mg L^-1^* |
| *Total phosphorus (PO_4_)* |  | *0.009* | *mg L^-1^* |
|  |  |  |  |
| Titrimetry | DIN 38406 E3-3; DIN 38409  H6; DIN 38409 H7 |  |  |
|  |  |  |  |
| *Alkalinity (pH=4.5)* |  | *0.09* | *mmol H+ L^-1^* |
| *Carbonate hardness* |  | *0.25* | *GRAD dH* |
| *Carbonate hardness (mmol L^-1^)* |  | *0.09* | *mmol L^-1^* |
|  |  |  |  |
| Photometry | DIN 38405 |  |  |
| *Dissolved silica (H_4_SiO_4_)* |  | *0.35* | *mg L^-1^* |
| *Dissolved silica (Si)* |  | *0.1* | *mg L^-1^* |
| *Dissolved silica (SiO_2_)* |  | *0.2* | *mg L^-1^* |
